# Heart donation and transplant recipient survival outcomes from deceased organ donors managed in hospital-based vs independent donor care units

**DOI:** 10.1016/j.healun.2025.02.1694

**Published:** 2025-03-03

**Authors:** Felicia Y. Ho, Xingmei Wang, Douglas E. Schaubel, Mauer Biscotti, Marisa Cevasco, Aditya G. Parikh, Meeta P. Kerlin, Jason D. Christie, Peter P. Reese, Emily A. Vail

**Affiliations:** a University of Pennsylvania Perelman School of Medicine, Philadelphia, Pennsylvania; b Department of Biostatistics, Epidemiology, and Informatics, University of Pennsylvania Perelman School of Medicine, Philadelphia, Pennsylvania; c Division of Cardiac Surgery, Department of Surgery, University of Pennsylvania Perelman School of Medicine, Philadelphia, Pennsylvania; d Division of Cardiology, Department of Internal Medicine, University of Pennsylvania Perelman School of Medicine, Philadelphia, Pennsylvania; e Division of Pulmonary, Allergy, and Critical Care, Department of Internal Medicine, University of Pennsylvania Perelman School of Medicine, Philadelphia, Pennsylvania; f Palliative and Advanced Illness Research (PAIR) Center, Perelman School of Medicine, University of Pennsylvania, Philadelphia, Pennsylvania; g Leonard Davis Institute for Health Economics, University of Pennsylvania, Philadelphia, Pennsylvania; h Vanderbilt Center for Transplant Science, Vanderbilt University Medical Center, Nashville, Tennessee; i Department of Anesthesiology and Critical Care, University of Pennsylvania Perelman School of Medicine, Philadelphia, Pennsylvania

**Keywords:** donation after brain death, organ donation, heart transplantation, donor care units, United States

## Abstract

**BACKGROUND:**

Despite growing prevalence, the impact of centralized deceased organ donor care units on heart donation and transplant recipient outcomes remains unknown.

**METHODS:**

We conducted a retrospective study using Organ Procurement and Transplantation Network deceased donor and transplant recipient data. We included adult deceased organ donors after brain death managed in 21 US regions with donor care units (DCUs) January 2019 to December 2022 and their heart recipients. The primary exposure was organ recovery in independent vs hospital-based DCUs. Outcomes included heart graft survival duration (primary) and incidence of heart donation.

**RESULTS:**

The cohort captured 9,830 donors after brain death, 3,302 in 10 independent DCUs (33.6%), and 1,366 (13.9%) in 11 hospital-based DCUs (the remainder in hospitals). Unadjusted heart donation did not differ between independent and hospital-based DCUs (40.9% vs 40.4%, *p* = 0.75). In a logistic regression model including donor factors, odds of heart donation did not differ by DCU type (adjusted odds ratio [aOR] 1.02, 95% confidence interval [CI] 0.86-1.22). Among 3,108 heart transplant recipients from cohort heart donors, restricted mean survival time 4 years after transplant was not different between hearts recovered in independent vs hospital-based DCUs (1,292 vs 1,276 days). In a Cox model including donor and recipient factors, adjusted hazards of graft failure were not different by DCU type (adjusted hazard ratio [aHR] 0.79, 95% CI 0.50-1.24).

**CONCLUSIONS:**

Among deceased organ donors after brain death, heart donation and heart transplant survival did not vary between hospital-based and independent DCUs. Further work is needed to determine if DCU utilization increases the number of hearts available for transplant.

## Background

Despite the ongoing expansion of organ donor eligibility criteria,^1,2^ adoption of normothermic regional perfusion in heart donation after circulatory death,^3^ and advances in heart preservation and storage technologies^4^ that have increased the pool of eligible donors and improved the quality and availability of hearts for transplantation, there remains a critical shortage.^5^ In the United States, one ongoing effort to expand access to transplantable organs is centralizing deceased organ donor management into regional donor care units (DCUs).^5,6^

While concentrated donor management expertise and resources in DCUs may plausibly improve organ donation rates from eligible donors, past work focused on independent DCUs (separate from acute-care hospitals) has yet to demonstrate significant differences in unadjusted heart donation rates (over traditional hospitals).^7^ One limitation of these DCUs may be the absence of specific heart donor assessment and management resources, such as coronary catheterization labs and licensed hospital beds, that may delay or preclude transfers of some potential heart donors (including donors after circulatory death [DCD]) to these facilities. However, most DCUs recently established in the United States are colocated within hospitals—potentially offering crucial shared resources for heart donor assessment and management.^6^

Recent work demonstrated differences in lung donation and transplant recipient survival between independent and hospital-based DCUs.^8^ Therefore, this study aimed to compare the duration of graft survival among deceased donors after brain death managed and recovered in hospital-based vs independent DCUs, and secondarily, to compare those outcomes between DCUs and acute-care hospitals. As unmodifiable donor and recipient factors may influence survival outcomes, we hypothesized that the duration of graft survival would be similar between hearts recovered from hospital-based and independent DCUs.

## Materials and methods

We conducted a retrospective cohort study of pre-existing data captured in the Organ Procurement and Transplantation Network (OPTN) deceased donor registry and heart transplant recipient characteristics and outcomes files, which includes all deceased organ donors and lung transplant recipients in the United States. Organ procurement organization and transplant program staff collected study data, which were compiled into datasets managed and distributed by the OPTN (based on data as of January 1, 2024). The data reported here have been supplied by the United Network for Organ Sharing (UNOS) as the contractor for the OPTN. The interpretation and reporting of these data are the responsibility of the authors and do not represent the official policy of or interpretation by the OPTN or the US Government.

The cohort included all deceased donors after brain death who underwent organ recovery in the United States from January 1, 2019, to December 31, 2022, and were captured in the study dataset. We excluded donors in the US regions without an operating DCU (as previously defined)^7,8^ and donors less than 16 years old (unlikely to be transferred to a DCU). We excluded DCD donors because independent DCUs rarely manage these donors.^6^ After cohort creation, we linked donors’ records to all heart transplant recipients captured in the study dataset, excluding simultaneous multivisceral transplants. For recipients of more than 1 heart transplant, we only included outcomes of the first transplant performed during the study period.

### Variable definitions

As described previously, the primary exposure was donor management and organ recovery in independent vs hospital-based DCUs (“DCU type”).^8^ The secondary exposure was donor management in DCUs vs acute-care hospitals. Because organ procurement organizations operate only one type of DCU for donors after brain death,^6^ we further stratified acute-care hospitals according to the type of DCU available in their donor service areas.

Secondary donor management outcomes included the use of coronary artery catheterization, pulmonary artery catheterization, and medications (including levothyroxine, dobutamine, and vasopressin) during the donor management period (between diagnosis of brain death and organ recovery). Secondary donation outcomes included heart donation rate, the total number of organs donated, and specific organs transplanted from cohort heart donors. The primary outcome was the duration of heart transplant graft survival (defined as patient death or retransplantation). Secondary recipient outcomes included recipients’ hospital lengths of stay after transplant and rates of graft survival at 1 year.

The OPTN defined study variables. We classified etiologies of heart failure into 8 groups,^9^ identified the presence of mechanical circulatory support at transplant,^10^ and categorized left ventricular assist devices (LVADs) present at the time of transplantation as permanent or temporary. We defined recipients’ severity of illness according to UNOS status at the time of transplant; recipients listed before the 2018 policy change^11^ were consolidated into contemporary classes.^12^ We defined expanded criteria heart donors according to Bakhtiyar et al.^13^ Predicted heart masses of donors and recipients were calculated according to the International Society for Heart and Lung Transplantation formulas, with the degree of mismatch classified according to Tao et al.^14,15^ These definitions are included in Table S1.

### Statistical analysis

After cohort creation, we first compared demographic, anthropometric, and clinical characteristics associated with heart donation and heart donor management among cohort donors according to organ recovery location (DCU or hospital and type of DCU available) using chi-square, *t*-tests, and Wilcoxon’s rank-sum tests. Next, we compared unadjusted heart donation rates between groups. To account for observed differences in donor characteristics associated with heart donation between recovery sites, we created a logistic regression model estimating the odds of heart donation among cohort donors after brain death using covariates included in the Scientific Registry of Transplant Recipients model predicting the likelihood of heart donation.^16^ We then estimated adjusted odds of heart donation between DCU types and between each type of DCU and regional hospitals.

Next, we compared the characteristics of heart donors and heart transplant recipients according to heart recovery location using standard tests without adjustment. We estimated the proportions of the heart donors in each hospital and DCU that received heart-specific donor therapies and graft quality testing (including levothyroxine and coronary catheterization). We compared these proportions to median rates among cohort donor hospitals (donors not transferred) in donation regions with independent and hospital-based DCUs. We estimated unadjusted durations of graft survival (the primary outcome) and graft survival at 1 year after transplant using the Kaplan-Meier method, compared survival using the log-rank test, and then calculated restricted mean survival time at 4 years after transplant (1 year before the maximum duration of follow-up).^17^

We developed multivariable Cox regression models, including key donor and recipient covariates with clinical plausibility and strong unadjusted associations in univariate comparisons in the primary comparison cohort, to account for observed differences in donor and recipient characteristics associated with heart transplant survival. We included transplant program and transplant year as stratifying variables (using a STRATA statement in the SAS PHREG procedure) to account for changes in outcomes over time and differences in outcomes among transplant programs without assuming proportionality. The Supplement describes formal testing of the proportional hazards assumption. We also estimated adjusted graft survival in each comparison group at 1 year.

The final cohort size was limited by the number of donors and recipients captured in the study dataset. Missing data were not imputed, and variables with more than 10% missing values were not reported or included in adjusted analyses. Adjusted models were generated from complete case analyses. The level of significance was defined as *p* = 0.05; we did not adjust for multiple comparisons. The Penn Medicine Institutional Review Board (protocol #850256) reviewed the study and determined it exempt from human subjects research designation. The study adheres to the principles of the World Medical Association Statement on Organ and Tissue Donation,^18^ the Declaration of Helsinki,^19^ and the Declaration of Istanbul.^20^ It is reported according to Strengthening the Reporting of Observational Studies in Epidemiology (STROBE) guidelines for observational research.^21^ Analyses were performed using SAS 9.4 (Cary, NC).

## Results

**Figure 1:**
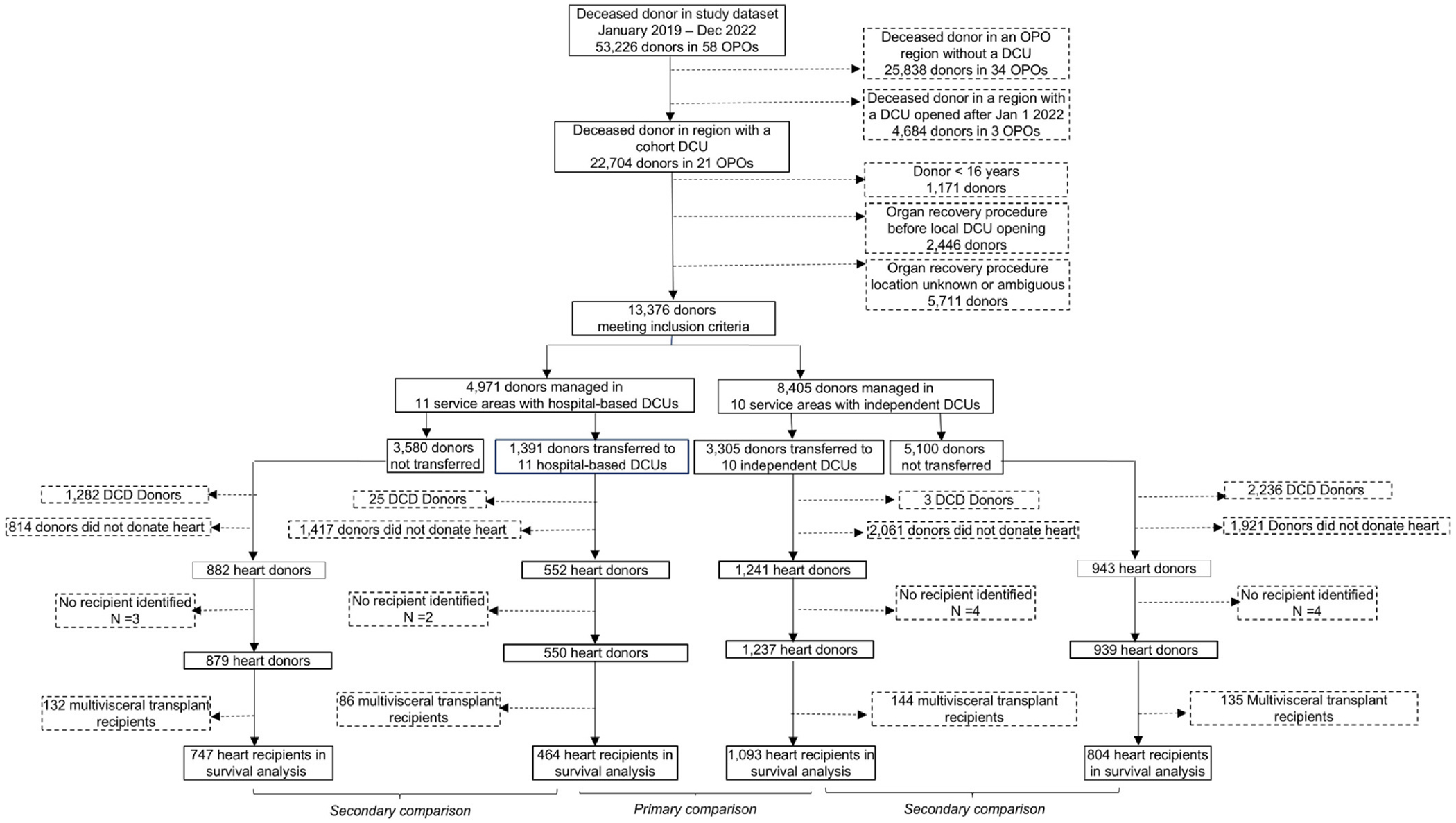
Cohort selection diagram. DCU, donor care unit. OPO, organ procurement organization.

After excluding DCD donors, the final cohort comprised 9,830 donors after brain death managed in 21 organ donation regions with operating DCUs (Figure 1). Of those donors, 3,302 were managed in 10 independent DCUs (33.6%) and 1,366 (13.9%) in 11 hospital-based DCUs; the remainder (5,162, 52.5%) were managed in hospitals. Characteristics and outcomes of donors managed in DCUs are presented in Table 1. Donors transferred to independent vs hospital-based DCUs had similar demographic, clinical, and management characteristics. Table S2 includes these comparisons for donors in DCUs vs hospitals.

The overall heart donation rate among cohort donors was 37.9%; unadjusted heart donation rates did not differ between donors managed in independent and hospital-based DCUs (40.9% vs 40.4%, *p* = 0.75). The only detectable difference in heart donation rate was between independent DCUs and hospitals in those regions (40.9% vs 32.9%, *p* < 0.001); heart donation rates were similar between hospital-based DCUs and regional hospitals (40.4% vs 38.4%, *p* = 0.22). Rates of heart donation varied among individual DCUs, from 28.8% to 50% among independent DCUs (median 37.1%) and 29.0% to 92.3% among hospital-based DCUs (median 43.4%) (Figure S1). Odds of heart donation were attenuated by adjustment. They did not vary between DCU types (adjusted odds ratio (aOR) 1.02, 95% confidence interval [CI] 0.86-1.22), between hospital-based DCUs and regional acute-care hospitals (aOR 1.17, 95% CI 0.95-1.45), or independent-based DCUs and hospitals (aOR 1.16, 95% CI 0.98-1.36).

Characteristics of heart donors managed in each DCU type are presented in Tables 2 and S3 (secondary comparisons). Compared to donors managed in independent DCUs, donors in hospital-based DCUs were similar in most demographic and clinical characteristics, including sex, age, and mechanism of death. Still, donors in hospital-based DCUs were more likely than those in independent DCUs to meet expanded heart donor criteria (183 [33.2%] vs 336 [27.1%] in independent DCUs, *p* = 0.01). Heart donors managed in hospital-based DCUs were significantly less likely to receive inotropes or vasopressin and more likely to receive levothyroxine than donors in independent DCUs. However, administration of these therapies varied substantially among individual DCUs (Figure S2). Heart donors had the shortest median donor management time (57 hours, range 46-76) in independent DCUs and the longest in hospital-based DCUs (70 hours, range 57-86; Table S4).

Among 3,108 heart transplant recipients in survival analyses, 2,245 (72.7%) were male; the median age at transplant was 55 (IQR 41-63). Among these patients, the median duration of follow-up was 729 days (range 374-1,807) overall and 735 days (range 45-1,807) among patients with censored outcomes. Over the observation period, 406 patients died (13.1% of the cohort), 17 underwent repeat transplants (0.6%), and 6 (0.2%) were lost to follow-up. Recipients of hearts recovered from donors managed in hospital-based and independent DCUs had similar ages, etiologies of heart failure, incidence of LVAD use and hospitalization before transplant, and UNOS status (Table 3; refer Table S5 for secondary comparisons).

**Figure 2:**
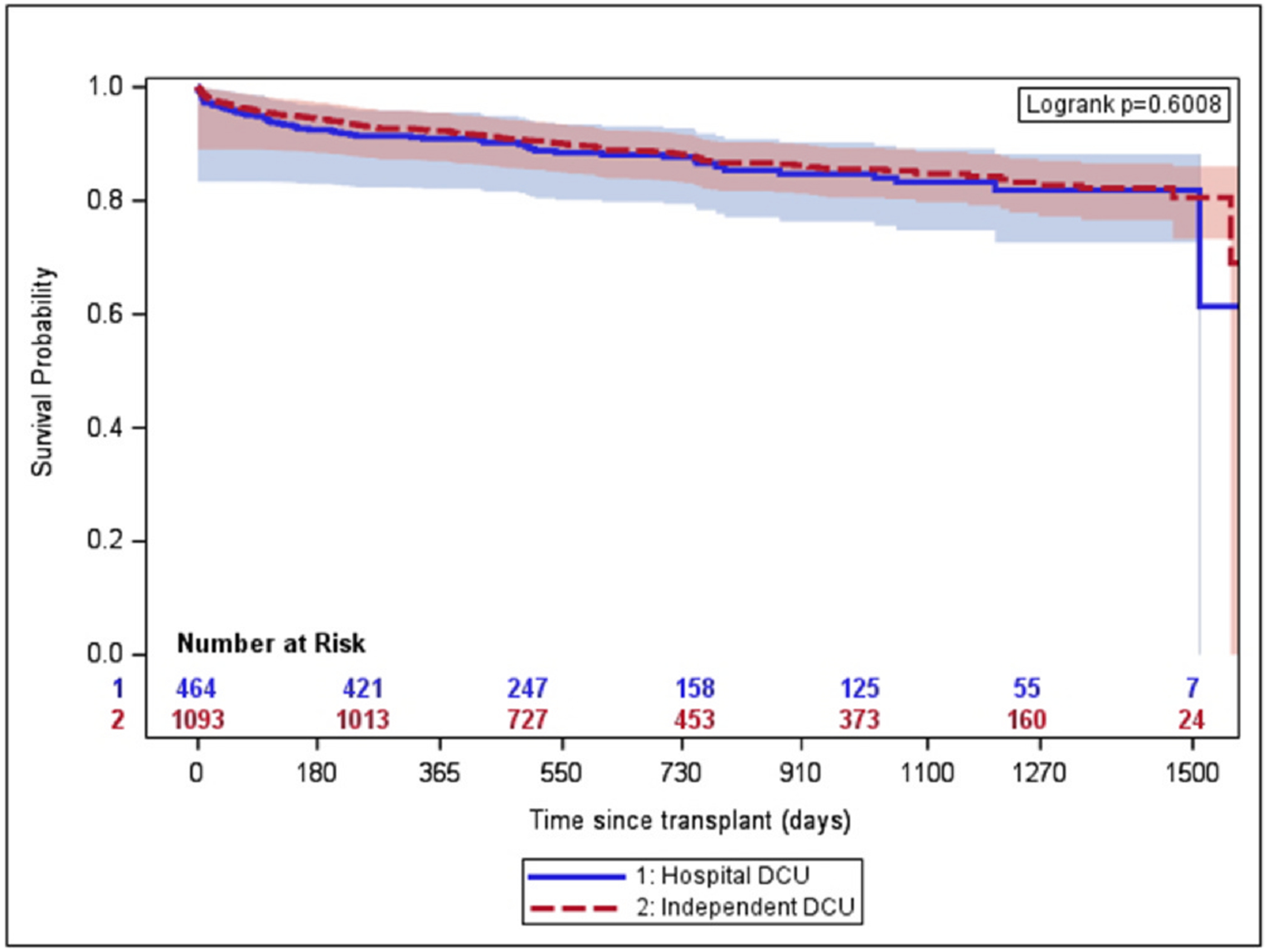
Unadjusted graft survival (independent vs hospital-based DCU recovered-donors). DCU, donor care unit. Blue line: grafts recovered from hospital-based donor care units. Red line: grafts recovered from independent donor care units. Shading represents 95% confidence intervals.

**Figure 3:**
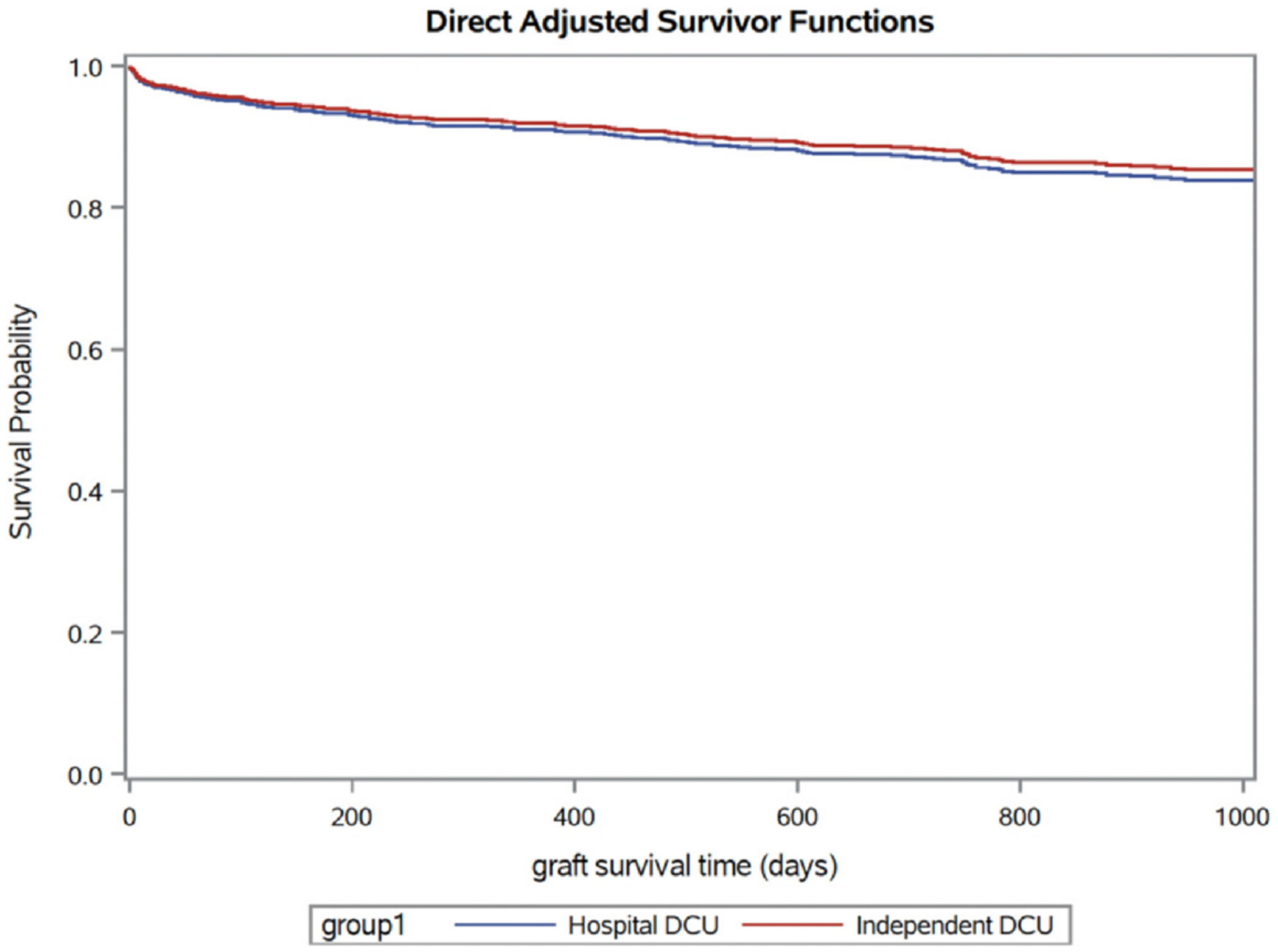
Adjusted survivor function curves. The blue line represents the estimated adjusted survival probability among hearts recovered from hospital-based DCUs and transplanted over time. The red line represents the estimated adjusted survival probability among heart grafts recovered and transplanted from independent DCUs. Curves adjusted for transplant year, transplant program, expanded criteria heart donor status, terminal left ventricular ejection fraction, recipient age, recipient sex, etiology of heart failure, UNOS status at listing, and mismatch between predicted donor and recipient heart mass (refer to Table S7 in the Supplement). DCU, donor care unit; UNOS, United Network for Organ Sharing.

There was no difference in unadjusted survival duration among recipients of donor hearts recovered in independent vs hospital-based DCUs (*p* = 0.60 by log-rank test, Figure 2). There were no differences in unadjusted survival between independent DCUs and hospitals in regions with operating independent DCUs (*p* = 0.24) or between hospital-based DCUs and hospitals in those regions (*p* = 0.77; Figure S3). After adjustment, the proportionality assumption was satisfied (Figure S4). Adjusted hazards of graft failure were not significantly different between grafts recovered from independent vs hospital-based DCUs (adjusted hazard ratio (aHR) 0.79, 95% CI 0.50-1.24; Figure 3 and Tables S7, S8). No differences were detected in adjusted graft failure models of secondary comparisons (aHR 0.87 (95% CI 0.63-1.20) for independent DCUs vs regional hospitals; aHR 1.46 (95% CI 0.93-2.28) between hospital-based DCUs and regional hospitals; Tables S9 and S10).

Secondary recipient outcomes, including unadjusted restricted mean survival time, hospital lengths of stay after transplant, and estimated unadjusted and adjusted graft survival at 1 year, were also similar between DCU types (Table 4). In secondary comparisons, there were no differences for 1-year graft survival (before or after adjustment) or restricted mean survival time at 4 years between groups (Table S6).

## Discussion

In a large retrospective cohort of heart donors and recipients managed in US regions with operating DCUs, the duration of heart transplant graft survival did not differ between organs recovered in independent vs hospital-based DCUs. Furthermore, apparent differences in heart donation rates between groups were attenuated by adjustment.

This builds on past work examining rates of heart donation in hospitals vs independent DCUs, including analyses of a smaller, earlier sample from the same study dataset, which found no significant differences in heart donation rates between recovery locations but lower donation rates (36.2%) overall.^7^ The study is consistent with other work demonstrating the longest donor management times in hospital-based DCUs and shortest (vs acute-care hospitals) in independent DCUs.^22,23^ Shorter donor management times may offer potential benefits for clinically unstable donors and recipients (who may develop complications and lose eligibility for donation or transplantation in the interim, respectively). These times are also commonly invoked markers of donation process efficiency and donor management cost savings (by reducing organ procurement organization (OPO) staffing hours and donor hospital lengths of stay). Conversely, undue emphasis on reducing donor management times may overlook opportunities to re-evaluate organ function serially or to allocate difficult-to-place organs. In the case of heart donation, specifically, additional donor management time may allow physiologic recovery from stress cardiomyopathy induced by sympathetic storm after brain death.^24^ In 1 study using OPTN data, investigators found that donor management times longer than 3 days (vs shorter periods) were associated with improved heart transplant recipient survival.^25^

This study is consistent with past observational studies demonstrating variation in donor management practices and outcomes among individual hospitals and OPOs in the United States and Canada.^26–29^ We found highly variable use of tests and therapies specific to heart donor quality assessment (including pulmonary artery and coronary catheterization) and donor management across hospitals and DCUs (including vasopressors, inotropes, and levothyroxine, a therapy with diminishing evidence of efficacy).^30^ However, we recognize that some recorded tests and interventions reflect donor management before DCU transfer, heterogeneity of individual donors, usual DCU processes, and transplant program requests.

Unlike recent work demonstrating a survival difference between lungs recovered in hospital-based vs independent DCUs,^8^ we did not observe differences in durations of heart survival across recovery locations (before or after adjustment for donor and recipient factors). One possibility is that the quality of recovered hearts may be less modifiable by available therapies than lungs, which may be rehabilitated using alveolar recruitment, lung protective mechanical ventilation, frequent bronchoscopy, and prone positioning.^31–33^ In this study, observed substantial variability in donor management between DCU types, individual DCUs, and hospitals, donation and recipient outcomes was not different between groups, which supports this hypothesis. Another possibility is that short-term outcomes (such as primary graft dysfunction) more directly attributable to heart donor management (including DCU use) not available or insufficiently captured in the study dataset remain undetected.

While not examined in the current study, we recognize that some evolving practices in heart donor management may be uniquely facilitated in hospital-based DCUs. These include standardizing the management of potential DCD donors,^3,34^ a growing source of donated organs. DCD donation requires licensed hospital beds (not available in independent DCUs) before the withdrawal of life-sustaining therapy. Hospital-based DCUs may further expand DCD heart donation efforts by leveraging available clinical perfusionists, equipment, and operating rooms to deliver normothermic regional perfusion and ex vivo machine perfusion technologies.^3,4^

### Limitations

This study is limited by factors impacting research using secondary data and may be at specific risk of confounding by indication—that is, we cannot ascertain why some donors were transferred to DCUs and others were not. Although many donor characteristics were similar between these groups, we cannot assume that transfer patterns (which may vary according to regional health care resources) were similar among individual DCUs or DCU types. This may be particularly relevant for potential heart donors, in which factors precluding donor transport to a DCU (e.g., hemodynamic instability, distance from the donor hospital, donor family preference) may also reduce the likelihood of successful heart allocation and recovery. The study is also at risk of misclassification bias (as organ recovery location recording remains optional in the dataset). Although recording rates increased over the study period, the potential to bias outcome comparisons in unpredictable directions remains. In addition, while the study period reflects a period of active DCU adoption, it precedes more recent innovations in the clinical management of decompensated heart failure (e.g., newer forms of percutaneous mechanical circulatory support and disease-modifying therapies) likely to impact recipient outcomes.^35^ Although the study dataset captures thousands of donors and recipients, substantial heterogeneity among cohort donors and recipients increases the likelihood that the study is under-powered to detect a true difference between groups, particularly in secondary comparisons of specific DCU types vs hospitals.

## Conclusions

In a retrospective study of deceased organ donors after brain death, heart transplant recipient survival did not differ between the type of DCU used for heart recovery. Further work is needed to determine how to use and operate donor care units to mitigate the critical shortage of hearts and other organs maximally.

## Supplementary Material

Supplementary material [1]

## Figures and Tables

**Table 1:** Cohort Characteristics Associated with Eligibility for Heart Donation and Heart Donation Rates by DCU Type.

Organ recovery location	Hospital-based DCUN=1366	Independent DCUN=3302	P value
*Characteristics associated with heart donation*			
Age, mean ± SD	43.7 ± 14.9	42.7 ± 14.7	0.03
Sex, n (%)			
Female	520 (38.1)	1299 (39.3)	0.42
Male	846 (61.9)	2003 (60.7)	
Height (cm), median (IQR)	170 (163, 178)	173 (165, 180)	0.001
Weight (kg), median (IQR)	81.0(68.9, 95.0)	81.6(69.5, 96.0)	0.09
Race and ethnicity, n (%)			
American Indian or Alaska Native	7 (0.5)	10 (0.3)	< .0001
Asian	64 (4.7)	56 (1.7)	
Black	279 (20.4)	699 (21.2)	
Hispanic	282 (20.6)	421 (12.7)	
Multiracial	12 (0.9)	12 (0.4)	
Native Hawaiian or other Pacific Islander	2 (0.1)	2 (0.1)	
White	720 (52.7)	2102 (63.7)	
Mechanism of death, n (%)			
Asphyxiation	59 (4.3)	140 (4.2)	0.12
Blunt injury	218 (16.0)	554 (16.8)	
Cardiovascular	258 (18.9)	587 (17.8)	
Death from natural causes	47 (3.4)	106 (3.2)	
Drowning	9 (0.7)	15 (0.5)	
Drug intoxication	213 (15.6)	623 (18.9)	
Electrical	1 (0.1)	3 (0.1)	
Gunshot wound	131 (9.6)	307 (9.3)	
Intracranial hemorrhage/stroke	410 (30.0)	884 (26.8)	
Seizure	11 (0.8)	41 (1.2)	
Stab	1 (0.1)	4 (0.1)	
None of the above	8 (0.6)	38 (1.2)	
History of diabetes, n (%)			
0-5 years	72 (5.3)	143 (4.3)	0.62
6-10 years	39 (2.9)	80 (2.4)	
10+ years	69 (5.1)	179 (5.4)	
History of cocaine use, n (%)	266 (19.5)	720 (21.8)	0.30
History of other drug use, n (%)	679 (49.7)	1763 (53.4)	0.15
History of alcohol use, n (%)	269 (19.7)	712 (21.6)	0.16
History of cancer, n (%)	45 (3.3)	98 (3.0)	0.84
Proteinuria, n (%)	688 (50.4)	1808 (54.8)	0.13
PaO2: FiO2, mmHg, median (IQR)	188 (111, 426)	184 (115, 430)	0.94
Serum BUN, mg/dL	27(19, 43)	25 (15, 45)	0.78
Serum creatinine, mg/dL	1.1 (0.8, 2.1)	1.2 (0.8, 2.2)	0.74
Bloodstream infection, n (%)	187 (13.7)	476 (14.4)	0.52
Pulmonary infection, n (%)	969 (70.9)	2100 (63.6)	< .0001
Urine infection, n (%)	242 (17.7)	510 (15.4)	0.06
Risk factors for blood borne disease transmission, n (%)	301 (22.0)	821 (24.9)	0.04
Blood Type, n (%)			
A	469 (34.3)	1180 (35.7)	< .0001
B	178 (13.0)	422 (12.8)	
AB	40 (3.0)	101 (3.1)	
O	679 (49.7)	1599 (48.4)	
*Clinical management relevant to heart donation*			
Levothyroxine, n (%)	819 (60.0)	831 (25.2)	< .0001
Vasopressin, n (%)	897 (65.7)	2365 (71.6)	0.01
Pulmonary artery catheter placed, n (%)	36 (2.6)	88 (2.7)	0.94
Left ventricular ejection fraction recorded, n (%)	1072 (78.5)	2470 (74.8)	0.008
Left ventricular ejection fraction, %, median (IQR)^a^	60 (55, 65)	60 (55, 65)	< .0001
*Donation outcomes*			
Heart donation, n (%)	552 (40.4)	1241 (40.9)	0.75
Adjusted odds of heart donation,^b^ (95% CI)	1.02 (0.86-1.22)^c^		

Abbreviations: CI: confidence interval, DCU: donor care unit, FiO_2_: fraction of inspired oxygen, IQR: interquartile range, PaO_2_: partial pressure of arterial oxygen SD: standard deviation

a Among donors with recorded values.

b Adjusted for covariates included in the Scientific Registry of Transplant Recipients’ heart donor yield prediction model.^16^

c Reference group: donation in a hospital-based DCU.

**Table 2:** Characteristics of Cohort Heart Donors Managed in Donor Care Units

N (%) or median (IQR)	Hospital-based DCU N = 552	Independent DCU N = 1,241	*p*-value
*Demographic characteristics*			
Donation year			< 0.0001
2019	73 (13.2%)	223 (18.0%)	
2020	119 (21.6%)	313 (25.2%)	
2021	163 (29.5%)	405 (32.6%)	
2022	197 (35.7%)	300 (24.2%)	
Age group, years			0.10
< 40	407 (73.7%)	970 (78.2%)	
40-60	143 (25.9%)	269 (21.7%)	
> 60	2 (0.4%)	2 (0.2%)	
Sex			0.60
Female	174 (31.5%)	376 (30.3%)	
Male	378 (68.5%)	865 (69.7%)	
Race and ethnicity			< 0.0001
American Indian or Alaska Native	3 (0.5%)	4 (0.3%)	
Asian	16 (2.9%)	10 (0.8%)	
Black	110 (19.9%)	243 (19.6%)	
Hispanic	125 (22.6%)	187 (15.1%)	
Multiracial	7 (1.3%)	9 (0.7%)	
White	291 (52.7%)	788 (63.5%)	
History of cancer	7 (1.3%)	18 (1.5%)	0.88
History of alcohol use	97 (17.6%)	220 (17.7%)	0.44
History of smoking	51 (9.2%)	152 (12.2%)	0.17
History of intravenous drug use	81 (14.7%)	244 (19.7%)	0.03
History of other drug use	332 (60.1%)	799 (64.4%)	0.41
History of diabetes			0.58
0-5 years	10 (1.8%)	21 (1.7%)	
6-10 years	3 (0.5%)	6 (0.5%)	
10+ years	9 (1.6%)	9 (0.7%)	
History of coronary artery disease	1 (0.2%)	6 (0.5%)	0.61
Hypertension	73 (13.2%)	170 (13.7%)	0.89
Received cardiopulmonary resuscitation (before brain death)	298 (54.0%)	674 (54.3%)	0.97
*Clinical characteristics*			
Height (cm)	173 (165, 180)	175 (168, 181)	0.006
Weight (kg)	80.5 (69.9, 93.9)	80.1 (70.0, 94.0)	0.61
Body mass index (kg/m^2^)	26.8 (23.7, 31.3)	26.4 (23.2, 30.7)	0.48
EBV IgG positive	502 (90.9%)	1,147 (92.4%)	0.001
EBV IgM positive	7 (1.3%)	16 (1.3%)	0.59
Mechanism of death			0.36
Asphyxiation	33 (6.0%)	67 (5.4%)	
Blunt injury	133 (24.1%)	291 (23.4%)	
Cardiovascular	61 (11.1%)	107 (8.6%)	
Death from natural causes	15 (2.7%)	28 (2.3%)	
Drowning	7 (1.3%)	8 (0.6%)	
Drug intoxication	119 (21.6%)	325 (26.2%)	
Electrical	1 (0.2%)	2 (0.2%)	
Gunshot wound	101 (18.3%)	239 (19.3%)	
Intracranial hemorrhage/stroke	70 (12.7%)	135 (10.9%)	
Seizure	8 (1.4%)	20 (1.6%)	
Stab	0 (0.0%)	4 (0.3%)	
None of the above	4 (0.7%)	15 (1.2%)	
Bloodstream infection	66 (12.0%)	144 (11.6%)	0.83
*Heart donor-specific management*			
Coronary angiogram performed	221 (40.0%)	555 (44.7%)	0.21
Pulmonary artery catheter inserted	17 (3.1%)	53 (4.3%)	0.28
Levothyroxine administered	302 (54.7%)	339 (27.3%)	< 0.0001
Inotropes received within 24 hours of aortic cross-clamp	154 (27.9%)	428 (34.5%)	0.02
Vasopressin received within 24 hours of aortic cross-clamp	375 (67.9%)	971 (78.2%)	0.0005
Dobutamine administered at procurement	1 (0.2%)	51 (4.7%)	< 0.0001
Dopamine administered at procurement	15 (3.2%)	35 (3.2%)	0.98
Norepinephrine administered at procurement	37 (8.0%)	225 (20.6%)	< 0.0001
Terminal left ventricular ejection fraction, %	64 (59, 66)	60 (57, 65)	< 0.0001
Expanded criteria donor^a^	183 (33.2%)	336 (27.1%)	0.01

Abbreviations: DCU, donor care unit; EBV, Epstein-Barr virus; IQR, interquartile range.

a Defined by Bakhtiyar et al.^13^

**Table 3:** Cohort Transplant Recipient Characteristics by Heart Recovery Location

N (%) or median (IQR)	Hospital DCU N = 464	Independent DCU N = 1,093	*p*-value
Heart transplant recipient program size (number of heart transplants performed during study period)			
First quartile (< 50)	12 (2.6%)	52 (4.8%)	0.26
Second quartile (50-109)	58 (12.5%)	140 (12.8%)	
Third quartile (109-183)	136 (29.3%)	316 (28.9%)	
Fourth quartile (≥183)	258 (55.6%)	585 (53.5%)	
Transplant year			
2019	66 (14.2%)	211 (19.3%)	< 0.0001
2020	94 (20.3%)	277 (25.3%)	
2021	138 (29.7%)	349 (31.9%)	
2022	166 (35.8%)	256 (23.4%)	
Age, years	56.0 (42.0, 63.0)	55.0 (42.0, 63.0)	0.91
Sex			
Female	134 (28.9%)	317 (29.0%)	0.96
Male	330 (71.1%)	776 (71.0%)	
Race and ethnicity			
American Indian or Alaska Native	0 (0.0%)	2 (0.2%)	0.03
Asian	23 (5.0%)	36 (3.3%)	
Black	131 (28.2%)	239 (21.9%)	
Hispanic	64 (13.8%)	138 (12.6%)	
Multiracial	3 (0.6%)	7 (0.6%)	
Native Hawaiian or other Pacific Islander	0 (0.0%)	2 (0.2%)	
White	243 (52.4%)	669 (61.2%)	
*Clinical characteristics*			
Height (cm)	174 (165, 180)	173 (165, 180)	0.60
Weight (kg)	80.6 (67.6, 93.4)	83.0 (70.0, 95.8)	0.11
Body mass index (kg/m^2^)	26.4 (23.3, 30.4)	27.4 (23.9, 31.3)	0.01
Etiology of heart failure			
Complex congenital	28 (6.0%)	54 (4.9%)	0.20
Dilated cardiomyopathy	366 (78.9%)	859 (78.6%)	
Hypertrophic cardiomyopathy	15 (3.2%)	41 (3.8%)	
Ischemic cardiomyopathy	10 (2.2%)	18 (1.6%)	
Valvular heart disease	3 (0.6%)	9 (0.8%)	
Restrictive cardiomyopathy	27 (5.8%)	46 (4.2%)	
Arrhythmogenic/right ventricular dysplasia	2 (0.4%)	13 (1.2%)	
Retransplant	4 (0.9%)	30 (2.7%)	
Other	0 (0.0%)	1 (0.1%)	
*Clinical characteristics at time of transplant*			
Systolic pulmonary artery pressure (mm Hg)	40 (30, 50)	38 (29, 50)	0.38
Mean pulmonary artery pressure (mm Hg) ± SD	28 ± 10.0	28 ± 10.0	0.56
Pulmonary capillary wedge pressure (mm Hg)	19 (12, 25)	17 (12, 24)	0.24
Cardiac index (liter/min/m^2^)	2.1 (1.8, 2.6)	2.1 (1.8, 2.5)	0.21
Pulmonary vascular resistance (WU)	2.1 (1.3, 2.9)	2.0 (1.3, 2.9)	0.85
Glomerular filtration rate (ml/min)^a^	85 (65, 112)	83 (64, 111)	0.37
Serum bilirubin (mg/dl)	0.7 (0.5, 1.1)	0.7 (0.4, 1.0)	0.41
Panel reactive antibodies	0.0 (0.0, 16.0)	0.0 (0.0, 7.0)	0.24
Epstein-Barr virus positive	401 (86.4%)	950 (86.9%)	0.76
*Clinical support at time of transplant*			
Hospitalization status			
Not hospitalized	140 (30.2%)	345 (31.6%)	0.82
Hospitalized, not in ICU	60 (12.9%)	145 (13.3%)	
Hospitalized, in ICU	264 (56.9%)	603 (55.2%)	
Mechanical ventilation	16 (3.4%)	26 (2.4%)	0.23
Vasoactive medications	222 (47.8%)	490 (44.8%)	0.57
Pulmonary vasodilator medications	43 (9.3%)	91 (8.3%)	0.83
Diuretic medications	311 (67.0%)	731 (66.9%)	0.29
Mechanical circulatory support^b^	146 (31.5%)	391 (35.8)	0.10
Type of LVAD^c^			
None	326 (70.3%)	723 (66.1%)	0.38
Permanent	106 (22.8%)	273 (25.0%)	
Temporary	17 (3.7%)	48 (4.4%)	
Other	15 (3.2%)	49 (4.5%)	
Severity of illness^d^			
Consolidated 1A	360 (77.6%)	840 (76.9%)	0.71
Consolidated 1B	83 (17.9%)	202 (18.5%)	
Consolidated 2	21 (4.5%)	48 (4.4%)	
Inactive	0 (0.0%)	3 (0.3%)	
*Transplant characteristics*			
Ex vivo machine perfusion	11 (2.4%)	11 (1.0%)	0.03
Total ischemic time (hours)	3.6 (3.0, 4.0)	3.5 (3.0, 4.1)	0.46
Gender mismatch, *n* (%)			
Donor female—recipient male	49 (10.6%)	128 (11.7%)	0.63
Donor male—recipient female	42 (9.1%)	117 (10.7%)	
Donor and recipient male	281 (60.6%)	648 (59.3%)	
Donor and recipient female	92 (19.8%)	200 (18.3%)	
PHM mismatch, percent (mean ± SD)^e^	5.2 ± 19.2	4.7 ± 18.7	0.61
PHM mismatch ratio category, *n* (%)			
Heart undersized (donor mass ≤86% of recipient)	58 (12.5%)	128 (11.7%)	0.90
Matched (within 87% and 114% of recipient)	296 (63.8%)	701 (64.1%)	
Heart oversized (donor mass > 114% of recipient)	110 (23.7%)	264 (24.2%)	
Cytomegalovirus status			
Donor positive/recipient negative	122 (26.3%)	294 (26.9%)	0.001
Donor and recipient positive	193 (41.6%)	391 (35.8%)	
Donor and recipient negative	67 (14.4%)	191 (17.5%)	
Donor negative/recipient positive	70 (15.1%)	210 (19.2%)	

Abbreviations: DCU, donor care unit; ICU, intensive care unit; PHM, predicted heart mass; LVAD, ventricular assist device; SD, standard deviation; UNOS, United Network for Organ Sharing; WU, Woods units.

a Calculated using Cockcroft-Gault formula.

b Includes right or left ventricular assist device, biventricular assist device, and total artificial heart. No recipients were supported with extracorporeal membrane oxygenation (ECMO) at time of transplant.

c Refer Table S1 for definitions.

d Grouped severity of illness categories based on UNOS status.^12^

e Calculated using the International Society for Heart and Lung Transplantation 2019 predicted heart mass calculator.^14^

**Table 4:** Secondary Cohort Transplant Recipient Outcomes by Heart Recovery Location

Organ recovery location	Hospital-based DCU N = 464	Independent DCU N = 1,093	*p*-value
*In-hospital outcomes*			
Length of stay after transplant (days), median (IQR)	17 (12, 26)	17 (12, 26)	0.97
Acute rejection before hospital discharge, *n* (%)	80 (17.2)	190 (17.4)	0.95
Dialysis before hospital discharge, *n* (%)	68 (14.7)	164 (15.0)	0.96
*One-year outcomes*			
Graft survival, %^a,b^	90.9	92.3	0.60
Adjusted graft survival,^c^% (95% CI)	91.8 (89.6-94.0)	91.8 (90.3-93.3)	0.04
*Long-term outcomes*			
4-year restricted mean survival time,^b^ days	1,276	1,292	

Abbreviations: CI, confidence interval; DCU, donor care unit; IQR, interquartile range.

a Estimated by the Kaplan-Meier method at 339 and 346 days (respectively).

b Unadjusted.

c Estimated from Cox proportional hazards models (refer Table S7 for covariates).

## Data Availability

The data reported here have been supplied by the United Network for Organ Sharing as the contractor for the Organ Procurement and Transplantation Network (OPTN). The interpretation and reporting of these data are the responsibility of the authors and in no way should be seen as an official policy of or interpretation by the OPTN or the US Government.
