# Supplementary material for: Heart donation and transplant recipient survival outcomes from deceased organ donors managed in hospital-based vs independent donor care units
Source: J Heart Lung Transplant. Author manuscript; Available in PMC 2025 Jul 29. (PMC12303759; doi:10.1016/j.healun.2025.02.1694)
Supplement: Supplementary material [1] [file NIHMS2079593-supplement-Supplementary_material__1_.pdf]

SUPPLEMENTAL MATERIAL

Heart donation and transplant recipient survival outcomes from deceased organ donors managed in hospital-based versus independent donor care units

Ho F et al. ***Journal of Heart and Lung Transplantation*** **2025**

TABLE OF CONTENTS

Table S1. Variable definitions 2

Supplemental Methods. Proportional hazards testing for the Cox survival model 3

Table S2. Secondary Comparisons. Cohort donor characteristics associated with eligibility for heart donation and heart donation rates in DCUs versus hospitals 4

Table S3. Secondary Comparisons. Characteristics of cohort heart donors managed in DCUs versus hospitals 6

Table S4. Organ donation processes and outcomes among heart donors by organ recovery site... 8

Table S5. Secondary Comparisons. Characteristics of cohort heart transplant recipients of hearts recovered from DCUs versus hospitals 9

Table S6. Secondary Comparisons. Secondary heart transplant recipient outcomes between organs recovered in DCUs versus hospitals 12

Table S7. Primary Comparison. Unadjusted and adjusted Cox models for heart graft failure among deceased heart donors after brain death managed in donor care units 13

Table S8. Primary comparison. Characteristics and unadjusted outcomes of heart transplant recipients included, versus excluded, from the adjusted Cox graft survival model due to missingness of at least one covariate 15

Table S9. Secondary Comparison. Adjusted Cox model for heart graft failure from deceased heart donors after brain death managed in hospitals versus regional hospital-based donor care units.. 17

Table S10. Secondary Comparison. Adjusted Cox model for heart graft failure from deceased heart donors after brain death managed in hospitals versus regional independent donor care units...... 18

Figure S1. Heart donation rates by recovery location 19

Figure S2. Variation in clinical donor management among cohort DCUs and hospitals 20

Figure S3. Secondary Comparisons. Unadjusted survival curves between recipients of hearts recovered from donor care units (DCUs) and hospitals, stratified by DCU type 21

Figure S4. Primary Comparison. Tests of the proportional hazards assumption for the Cox survival model comparing grafts recovered from hospital-based versus independent DCUs 22

Figure S5. Secondary Comparison. Adjusted survivor function curves between DCUs and hospitals (stratified by DCU type available) 23

References 25

Table S1. Variable definitions

Variable	Reference	Category level	OPTN variable definition
Etiology of heart failure	Sorabella et al.[1]	Complex Congenital	TCR_DGN = 1203, 1205-7, 1500-2, 1548, 1549
		Dilated Cardiomyopathy	1000-10, 1049
		Hypertrophic Cardiomyopathy	1201
		Ischemic Cardiomyopathy	1200
		Restrictive Cardiomyopathy	1050-4, 1099
		Retransplant	1100-6, 1199
		Arrhythmogenic right ventricular dysplasia/cardiomyopathy	1208
		Valvular Heart Disease	1202
		Other	1204, 1209, 1497
Expanded criteria donor	Bakhityar et al.[2]	Donor age	AGE_DON
		Donor race and ethnicity	ETHCAT_DON
		Donor BUN	BUN_DON
		Donor creatinine	CREAT_DON
		Recipient race and ethnicity	ETHCAT
		Ischemic time	ISCHTIME
Type of mechanical circulatory support	Whitbread et al.[3]	Any support	VAD_DEVICE_TY_TRR = 2,3,4,5
Type of left ventricular assist device	Author consensus	None	VAD_DEVICE_TY_TRR = 1
		Temporary	VAD_DEVICE_TY_TRR = 2 AND VAD_BRAND1_TRR = 215, 217, 222, 225-8, 234, 235, 237, 238, 310, 311, 313, 316, 318, 320, 328, 329, 331, 332, 402
		Permanent	VAD_DEVICE_TY_TRR = 2 AND VAD_BRAND1_TRR = 205, 208, 209, 210, 223, 224, 236, 330
		Other	VAD_DEVICE_TY_TRR = 2 AND VAD_BRAND1_TRR = 241, 999

OPTN: Organ Procurement and Transplantation Network

Supplemental Methods. Proportional hazards testing for the Cox survival model

We formally tested the proportional hazards assumption by plotting baseline survivor function curves over time for each DCU type with model covariates fixed at central values. Next, we plotted the log cumulative hazards function (log (-log) of the survival function curves) of the adjusted model for each DCU type over time. Last, we created a secondary model including an interaction term for DCU type and follow-up time (allowing the DCU effect to change monotonically over time). Diagnostic plots are included in this Supplement (Figure S4).

Table S2. Secondary comparisons. Cohort donor characteristics associated with eligibility for heart donation and heart donation rates in DCUs versus hospitals

**Type of DCU available**	**Hospital-based**			**Independent**		
**Organ recovery location**	**DCU**	**Hospital**	**P-value**	**DCU**	**Hospital**	**P-value**
Organ donors, n (% of cohort)	1366 (13.9)	2298 (23.4)		3302 (33.6)	2864 (29.1)	
Characteristics associated with heart donation						
Age, mean ± SD	43.7 ± 14.9	42.5 ±15.1	0.02	42.7 ± 14.7	43.3 ± 15.8	0.09
Sex, n (%)						
Female	520 (38.1)	888 (38.6)	0.73	1299 (39.3)	1090 (38.1)	0.30
Male	846 (61.9)	1410 (61.4)		2003 (60.7)	1774 (61.9)	
Height (cm), median (IQR)	170 (163, 178)	172 (164, 178)	0.46	173 (165, 180)	170 (163, 178)	<.0001
Weight (kg), median (IQR)	81.0 (68.9, 95.0)	80.5 (69.6, 95.2)	0.83	81.6 (69.5, 96.0)	81.0 (68.6, 96.2)	0.42
Race and ethnicity, n (%)						
American Indian or Alaska Native	7 (0.5)	19 (0.8)	0.02	10 (0.3)	9 (0.3)	<.0001
Asian	64 (4.7)	107 (4.7)		56 (1.7)	115 (4.0)	
Black	279 (20.4)	366 (15.9)		699 (21.2)	465 (16.2)	
Hispanic	282 (20.6)	524 (22.8)		421 (12.7)	652 (22.8)	
Multiracial	12 (0.9)	25 (1.1)		12 (0.4)	28 (1.0)	
Native Hawaiian or other Pacific Islander	2 (0.1)	7 (0.3)		2 (0.1)	6 (0.2)	
White	720 (52.7)	1250 (54.4)		2102 (63.7)	1589 (55.5)	
Mechanism of death, n (%)						
Asphyxiation	59 (4.3)	97 (4.2)	0.08	140 (4.2)	113 (3.9)	<.0001
Blunt injury	218 (16.0)	414 (18.0)		554 (16.8)	517 (18.1)	
Cardiovascular	258 (18.9)	386 (16.8)		587 (17.8)	526 (18.4)	
Death from natural causes	47 (3.4)	110 (4.8)		106 (3.2)	46 (1.6)	
Drowning	9 (0.7)	9 (0.4)		15 (0.5)	11 (0.4)	
Drug intoxication	213 (15.6)	306 (13.3)		623 (18.9)	400 (14.0)	
Electrical	1 (0.1)	0 (0.0)		3 (0.1)	4 (0.1)	
Gunshot wound	131 (9.6)	220 (9.6)		307 (9.3)	255 (8.9)	
Intracranial hemorrhage/stroke	410 (30.0)	705 (30.7)		884 (26.8)	926 (32.3)	
Seizure	11 (0.8)	19 (0.8)		41 (1.2)	24 (0.8)	
Stab	1 (0.1)	5 (0.2)		4 (0.1)	8 (0.3)	
None of the above	8 (0.6)	27 (1.2)		38 (1.2)	34 (1.2)	
History of diabetes, n (%)						
0-5 years	72 (5.3)	118 (5.1)	0.96	143 (4.3)	148 (5.2)	0.08
6-10 years	39 (2.9)	56 (2.4)		80 (2.4)	76 (2.7)	
10+ years	69 (5.1)	107 (4.7)		179 (5.4)	131 (4.6)	
History of cocaine use, n (%)	266 (19.5)	425 (18.5)	0.74	720 (21.8)	562 (19.6)	0.03
History of other drug use, n (%)	679 (49.7)	1046 (45.5)	0.03	1763 (53.4)	1333 (46.5)	<.0001
History of alcohol use, n (%)	269 (19.7)	450 (19.6)	0.97	712 (21.6)	492 (17.2)	0.0001
History of cancer, n (%)	45 (3.3)	67 (2.9)	0.78	98 (3.0)	103 (3.6)	0.05
Proteinuria, n (%)	688 (50.4)	1282 (55.8)	0.003	1808 (54.8)	1419 (49.5)	<.0001
PaO2: FiO2, mmHg, median (IQR)	188 (111, 426)	159 (104, 392)	<.0001	184 (115, 430)	154 (109, 313)	<.0001
Serum BUN, mg/dL	27 (19, 43)	26 (18, 41)	0.04	25 (15, 45)	30 (17, 51)	<.0001
Serum creatinine, mg/dL	1.1 (0.8, 2.1)	1.1 (0.8, 2.1)	0.67	1.2 (0.8, 2.2)	1.3 (0.9, 2.3)	0.43
Bloodstream infection, n (%)	187 (13.7)	306 (13.3)	0.75	476 (14.4)	374 (13.1)	0.12

Type of DCU available	Hospital-based			Independent		
**Organ recovery location**	**DCU**	**Hospital**	**P-value**	**DCU**	**Hospital**	**P-value**
Pulmonary infection, n (%)	969 (70.9)	1644 (71.5)	0.70	2100 (63.6)	1790 (62.5)	0.37
Urine infection, n (%)	242 (17.7)	347 (15.1)	0.04	510 (15.4)	444 (15.5)	0.95
Risk factors for blood borne disease transmission, n (%)	301 (22.0)	513 (22.3)	0.84	821 (24.9)	659 (23.0)	0.09
Blood Type, n (%)						
A	469 (34.3)	792 (34.5)	0.52	1180 (35.7)	993 (34.7)	0.32
B	178 (13.0)	281 (12.2)		422 (12.8)	369 (12.9)	
AB	40 (3.0)	70 (3.0)		101 (3.1)	112 (3.9)	
O	679 (49.7)	1155 (50.3)		1599 (48.4)	1390 (48.5)	
Clinical management relevant to heart donation						
Levothyroxine, n (%)	819 (60.0)	1414 (61.5)	0.37	831 (25.2)	1159 (40.5)	<.0001
Vasopressin, n (%)	897 (65.7)	1516 (66.0)	0.94	2365 (71.6)	2148 (75.0)	0.02
Pulmonary artery catheter placed, n (%)	36 (2.6)	32 (1.4)	0.01	88 (2.7)	28 (1.0)	<.0001
Left ventricular ejection fraction recorded, n (%)	1072 (78.5)	1791 (77.9)	0.70	2470 (74.8)	2119 (74.0)	0.464
Left ventricular ejection fraction, %, median (IQR)[a]	60 (55, 65)	60 (55, 65)	0.004	60 (55, 65)	60 (55, 65)	0.001
Donation outcomes						
Heart donation, n (%)	552 (40.4)	882 (38.4)	0.22	1241 (40.9)	943 (32.9)	<.0001
Adjusted odds of heart donation,[b] (95% CI)	1.17 (0.95-1.45)[c]			1.16 (0.98-1.36)[c]		

CI: confidence interval, DCU: donor care unit, FiO2: fraction of inspired oxygen, IQR: interquartile range, PaO2: partial pressure of arterial oxygen SD: standard deviation

[a]Among donors with recorded values. [b]Adjusted for covariates included in the Scientific Registry of Transplant Recipients' heart donor yield prediction model.[4] [c]Reference group: donation in a hospital.

Table S3. Secondary comparisons. Characteristics of cohort heart donors managed in DCUs versus hospitals

**Type of DCU available**	**Hospital-based**			**Independent**		
**Organ recovery location**	**DCU N = 552**	**Hospital N = 882**	**P-value**	**DCU N = 1,241**	**Hospital N = 943**	**P-value**
Demographic characteristics						
Donation year, n (%)						
2019	73 (13.2)	99 (11.2)	0.0006	223 (18.0)	266 (28.2)	<.0001
2020	119 (21.6)	233 (26.4)		313 (25.2)	264 (28)	
2021	163 (29.5)	315 (35.7)		405 (32.6)	230 (24.4)	
2022	197 (35.7)	235 (26.6)		300 (24.2)	183 (19.4)	
Age group, years, n (%)						
<40	407 (73.7)	651 (73.8)	0.60	970 (78.2)	751 (79.6)	0.35
40-60	143 (25.9)	230 (26.1)		269 (21.7)	192 (20.4)	
>60	2 (0.4)	1 (0.1)		2 (0.2)	0 (0.0)	
Sex, n (%)						
Female	174 (31.5)	226 (25.6)	0.02	376 (30.3)	247 (26.2)	0.04
Male	378 (68.5)	656 (74.4)		865 (69.7)	696 (73.8)	
Race and ethnicity, n (%)						
American Indian or Alaska Native	3 (0.5)	11 (1.2)	0.02	4 (0.3)	2 (0.2)	<.0001
Asian	16 (2.9)	32 (3.6)		10 (0.8)	24 (2.5)	
Black	110 (19.9)	119 (13.5)		243 (19.6)	132 (14.0)	
Hispanic	125 (22.6)	234 (26.5)		187 (15.1)	252 (26.7)	
Multiracial	7 (1.3)	11 (1.2)		9 (0.7)	13 (1.4)	
Native Hawaiian/other Pacific Islander	0 (0.0)	3 (0.3)		0 (0.0)	3 (0.3)	
White	291 (52.7)	472 (53.5)		788 (63.5)	517 (54.8)	
History of cancer, n (%)	7 (1.3)	7 (0.8)	0.54	18 (1.5)	8 (0.8)	0.23
History of alcohol use, n (%)	97 (17.6)	153 (17.3)	0.77	220 (17.7)	143 (15.2)	0.28
History of smoking, n (%)	51 (9.2)	89 (10.1)	0.86	152 (12.2)	94 (10.0)	0.15
History of intravenous drug use, n (%)	81 (14.7)	99 (11.2)	0.10	244 (19.7)	138 (14.6)	0.003
History of other drug use, n (%)	332 (60.1)	491 (55.7)	0.08	799 (64.4)	572 (60.7)	0.01
History of coronary artery disease, n (%)	1 (0.2)	0 (0.0)	0.25	6 (0.5)	2 (0.2)	0.14
Hypertension, n (%)	73 (13.2)	120 (13.6)	0.53	170 (13.7)	114 (12.1)	0.19
History of Diabetes, n (%)						
0-5 years	10 (1.8)	14 (1.6)	0.33	21 (1.7)	18 (1.9)	0.37
6-10 years	3 (0.5)	6 (0.7)		6 (0.5)	8 (0.8)	
10+ years	9 (1.6)	5 (0.6)		9 (0.7)	6 (0.6)	
Received cardiopulmonary resuscitation (before brain death), n (%)	298 (54.0)	421 (47.7)	0.04	674 (54.3)	484 (51.3)	0.40
Clinical characteristics						
Height (cm), median (IQR)	173 (165, 180)	175 (168, 180)	0.01	175 (168, 181)	175 (168, 180)	0.55
Weight (kg), median (IQR)	80.5 (69.9, 93.9)	79.4 (70.0, 92.1)	0.66	80.1 (70.0, 94.0)	81.0 (69.7, 94.8)	0.79

Type of DCU available	Hospital-based			Independent		
**Organ recovery location**	**DCU N = 552**	**Hospital N = 882**	**P-value**	**DCU N = 1,241**	**Hospital N = 943**	**P-value**
Body mass index (kg/m2), median (IQR)	26.8 (23.7, 31.3)	26.2 (23.2, 30.3)	0.07	26.4 (23.2, 30.7)	26.8 (23.4, 30.8)	0.72
EBV IgG Positive, n (%)	502 (90.9)	809 (91.7)	0.58	1147 (92.4)	875 (92.8)	0.92
EBV IgM Positive, n (%)	7 (1.3)	12 (1.4)	0.14	16 (1.3)	23 (2.4)	<.0001
Mechanism of death, n (%)						
Asphyxiation	33 (6.0)	45 (5.1)	0.13	67 (5.4)	57 (6.0)	0.004
Blunt injury	133 (24.1)	235 (26.6)		291 (23.4)	252 (26.7)	
Cardiovascular	61 (11.1)	69 (7.8)		107 (8.6)	91 (9.7)	
Death from natural causes	15 (2.7)	27 (3.1)		28 (2.3)	11 (1.2)	
Drowning	7 (1.3)	5 (0.6)		8 (0.6)	2 (0.2)	
Drug intoxication	119 (21.6)	173 (19.6)		325 (26.2)	194 (20.6)	
Electrical	1 (0.2)	0 (0.0)		2 (0.2)	4 (0.4)	
Gunshot wound	101 (18.3)	156 (17.7)		239 (19.3)	167 (17.7)	
Intracranial hemorrhage/stroke	70 (12.7)	148 (16.8)		135 (10.9)	142 (15.1)	
Seizure	8 (1.4)	10 (1.1)		20 (1.6)	13 (1.4)	
Stab	0 (0.0)	2 (0.2)		4 (0.3)	4 (0.4)	
Other	4 (0.7)	12 (1.4)		15 (1.2)	6 (0.6)	
Bloodstream infection, n (%)	66 (12.0)	97 (11.0)	0.58	144 (11.6)	95 (10.1)	0.26
Heart donor-specific management						
Coronary angiogram, n (%)	221 (40.0)	330 (37.4)	0.23	555 (44.7)	390 (41.4)	0.06
Pulmonary artery catheter, n (%)	17 (3.1)	13 (1.5)	0.04	53 (4.3)	13 (1.4)	0.0001
Levothyroxine, n (%)	302 (54.7)	539 (61.1)	0.02	339 (27.3)	358 (38)	<.0001
Inotropes received within 24h of aortic cross-clamp, n (%)	154 (27.9)	307 (34.8)	0.01	428 (34.5)	366 (38.8)	0.09
Vasopressin received within 24h of aortic cross-clamp, n (%)	375 (67.9)	622 (70.5)	0.42	971 (78.2)	750 (79.5)	0.68
Dobutamine administered at procurement, n (%)	1 (0.2)	2 (0.2)	0.86	51 (4.1)	133 (14.1)	<.0001
Dopamine administered at procurement, n (%)	15 (2.7)	14 (1.6)	0.13	35 (2.8)	27 (2.9)	0.85
Norepinephrine administered at procurement, n (%)	37 (6.7)	72 (8.2)	0.33	225 (18.1)	92 (9.8)	<.0001
Terminal left ventricular ejection fraction (%), median (IQR)	64 (59, 66)	62 (58, 65)	0.16	60 (57, 65)	60 (57, 65)	0.70
Expanded criteria donor,[2] n (%)	183 (33.2)	315 (35.7)	0.32	336 (27.1)	309 (32.8)	0.004

DCU: donor care unit, EBV: Epstein-Barr virus, IQR: interquartile range

3605 (9.6% of cohort) heart donors had identifiable recipients included in survival analyses.

Table S4. Organ donation processes and outcomes among heart donors by organ recovery site

**Type of DCU available**	**Hospital-based**			**Independent**			***Primary comparison* Between DCU types p-value**
**Organ recovery location**	**DCU N=552**	**Hospital N=882**	**p-value**	**DCU N=1,241**	**Hospital N=943**	**p-value**	
Donor length of stay, days,[a] median (range)	6 (4-7)	5 (4-7)	0.19	5 (4-7)	5 (4-7)	0.93	0.61
Donor management time, hours,[b] median (range)	70 (57-86)	65 (51-81)	<.0001	57 (46-76)	67 (49-89)	<.0001	<.0001
Number of organs transplanted, median (IQR)	5 (4, 6)	4 (4, 6)	0.003	5 (4, 6)	5 (4, 6)	0.04	0.27
Lung donor, n (%)	263 (47.6)	339 (38.4)	0.001	610 (49.2)	431 (45.7)	0.11	0.56
Number of lungs transplanted, median (IQR)	0 (0, 2)	0 (0, 2)	0.0001	0 (0, 2)	0 (0, 2)	0.11	0.66
Liver donor, n (%)	509 (92.2)	796 (90.3)	0.21	1,134 (91.4)	851 (90.2)	0.36	0.56
Kidney donor, n (%)	522 (94.6)	833 (94.4)	0.92	1,176 (94.8)	899 (95.3)	0.54	0.86
Number of kidneys transplanted, median (IQR)	2 (2, 2)	2 (2, 2)	0.82	2 (2, 2)	2 (2, 2)	0.95	0.73
Pancreas donor, n (%)	91 (16.5)	158 (17.9)	0.49	260 (21.0)	157 (16.7)	0.01	0.03
Ex vivo machine perfusion (heart), n (%)	13 (2.4)	14 (1.6)	0.28	14 (1.1)	8 (0.8)	0.50	0.04

[a]Whole or partial calendar days. [b]Times less than the 1st percentile or greater than the 99th percentile excluded before comparison.

Table S5. Secondary comparisons. Characteristics of cohort transplant recipients with hearts recovered from DCUs versus hospitals

**Type of DCU available**	**Hospital-based**			**Independent**		
**Organ recovery location**	**DCU N = 464**	**Hospital N = 747**	**P-value**	**DCU N = 1,093**	**Hospital N = 804**	**P-value**
Heart transplant recipient program size (number of heart transplants performed during study period), n (%)						
First quartile (<50)	12 (2.6)	25 (3.3)	0.05	52 (4.8)	25 (3.1)	0.13
Second quartile (50 - 109)	58 (12.5)	111 (14.9)		140 (12.8)	91 (11.3)	
Third quartile (109 - 183)	136 (29.3)	167 (22.4)		316 (28.9)	259 (32.2)	
Fourth quartile (≥183)	258 (55.6)	444 (59.4)		585 (53.5)	429 (53.4)	
Transplant year, n (%)						
2019	66 (14.2)	83 (11.1)	0.0002	211 (19.3)	234 (29.1)	<.0001
2020	94 (20.3)	204 (27.3)		277 (25.3)	225 (28.0)	
2021	138 (29.7)	265 (35.5)		349 (31.9)	188 (23.4)	
2022	166 (35.8)	195 (26.1)		256 (23.4)	157 (19.5)	
Age (years), median (IQR)	56.0 (42.0, 63.0)	56.0 (41.0, 64.0)	1.00	55.0 (42.0, 63.0)	54.0 (40.5, 62.0)	0.12
Sex, n (%)						
Female	134 (28.9)	205 (27.4)	0.59	317 (29.0)	207 (25.7)	0.12
Male	330 (71.1)	542 (72.6)		776 (71.0)	597 (74.3)	
Race and ethnicity, n (%)						
American Indian or Alaska Native	0 (0.0)	3 (0.4)	0.04	2 (0.2)	4 (0.5)	0.07
Asian	23 (5.0)	50 (6.7)		36 (3.3)	40 (0.5)	
Black	131 (28.2)	167 (22.4)		239 (21.9)	166 (20.6)	
Hispanic	64 (13.8)	115 (15.4)		138 (12.6)	117 (14.6)	
Multiracial	3 (0.6)	4 (0.5)		7 (0.6)	10 (1.2)	
Native Hawai'ian or other Pacific Islander	0 (0.0)	8 (1.1)		2 (0.2)	5 (0.6)	
White	243 (52.4)	400 (53.5)		669 (61.2)	462 (57.5)	
Clinical characteristics						
Height (cm), median (IQR)	174 (165, 180)	173 (165, 180)	0.57	173 (165, 180)	173 (165, 180)	0.79
Weight (kg), median (IQR)	80.6 (67.6, 93.4)	80.4 (67.6, 92.8)	0.64	83.0 (70.0, 95.8)	80.7 (68.2, 93.9)	0.08
Body mass index (kg/m2)	26.4 (23.3, 30.4)	26.9 (23.5, 30.3)	0.94	27.4 (23.9, 31.3)	26.9 (23.3, 31.1)	0.04
Etiology of heart failure,[1] n (%)						
Complex Congenital	28 (6.0)	42 (5.6)	0.32	54 (4.9)	51 (6.3)	0.51
Dilated cardiomyopathy	366 (78.9)	576 (77.1)		859 (78.6)	633 (78.7)	
Hypertrophic cardiomyopathy	15 (3.2)	19 (2.5)		41 (3.8)	21 (2.6)	
Ischemic cardiomyopathy	10 (2.2)	10 (1.3)		18 (1.6)	16 (2.0)	
Valvular heart disease	3 (0.6)	9 (1.2)		9 (0.8)	12 (1.5)	
Restrictive cardiomyopathy	27 (5.8)	48 (6.4)		46 (4.2)	34 (4.2)	
Arrhythmogenic / right ventricular dysplasia	2 (0.4)	11 (1.5)		13 (1.2)	8 (1.0)	
Retransplant	4 (0.9)	16 (2.1)		30 (2.7)	17 (2.1)	
Other	0 (0.0)	1 (0.1)		1 (0.1)	0 (0.0)	
Clinical characteristics at time of transplant						
Systolic pulmonary artery pressure (mmHg), median (IQR)	40 (30, 50)	38 (29, 49)	0.10	38 (29, 50)	39 (29, 49)	0.39

Type of DCU available	Hospital-based			Independent		
**Organ recovery location**	**DCU N = 464**	**Hospital N = 747**	**P-value**	**DCU N = 1,093**	**Hospital N = 804**	**P-value**
Mean pulmonary artery pressure (mmHg) ± SD	28 ± 10.0	28 ± 10.0	0.63	27 ± 10	27 ± 10.0	0.96
Pulmonary capillary wedge pressure (mmHg), median (IQR)	19 (12, 25)	18 (12, 25)	0.21	17 (12, 24)	18 (12, 25)	0.26
Cardiac index (L/min/m2), median (IQR)	2.1 (1.8, 2.6)	2.1 (1.7, 2.5)	0.37	2.1 (1.8, 2.5)	2.1 (1.7, 2.5)	0.38
Pulmonary vascular resistance (WU), median (IQR)	2.1 (1.3, 2.9)	2.1 (1.4, 3.3)	0.18	2.0 (1.3, 2.9)	2.1 (1.3, 3.0)	0.36
Glomerular filtration rate (ml/min),[a] median (IQR)	85 (65, 112)	83 (64, 113)	0.43	83 (64, 111)	85 (65, 113)	0.08
Serum bilirubin (mg/dL), median (IQR)	0.7 (0.5, 1.1)	0.7 (0.5, 1.1)	0.45	0.7 (0.4, 1.0)	0.7 (0.4, 1.1)	0.83
Panel reactive antibodies, median (IQR)	0 (0, 16)	0 (0, 9.5)	0.55	0 (0, 7)	0 (0, 9)	0.99
Epstein-Barr virus positive, n (%)	401 (86.4)	638 (85.4)	0.96	950 (86.9)	668 (83.1)	0.03
Clinical support at time of transplant						
Hospitalization status, n (%)						
Not hospitalized	140 (30.2)	226 (30.3)	0.19	345 (31.6)	227 (28.2)	0.19
Hospitalized, Not in ICU	60 (12.9)	124 (16.6)		145 (13.3)	100 (12.4)	
Hospitalized, In ICU	264 (56.9)	397 (53.1)		603 (55.2)	477 (59.3)	
Mechanical ventilation, n (%)	16 (3.4)	7 (0.9)	0.002	26 (2.4)	27 (3.4)	0.20
Vasoactive medications, n (%)	222 (47.8)	362 (48.5)	0.83	490 (44.8)	378 (47.0)	0.44
Pulmonary vasodilator medications, n (%)	43 (9.3)	70 (9.4)	0.70	91 (8.3)	83 (10.3)	0.20
Diuretic medications, n (%)	311 (67.0)	532 (71.2)	0.16	731 (66.9)	524 (65.2)	0.67
Mechanical circulatory support,[b] n (%)	146 (31.5)	253 (33.9)	0.39	391 (35.8)	270 (33.6)	0.32
Type of LVAD,[c] n (%)						
None	326 (70.3)	505 (67.6)	0.40	723 (66.1)	556 (69.2)	0.40
Permanent	106 (22.8)	177 (23.7)		273 (25.0)	182 (22.6)	
Temporary	17 (3.7)	26 (3.5)		48 (4.4)	38 (4.7)	
Other	15 (3.2)	39 (5.2)		49 (4.4)	28 (3.5)	
Severity of illness at time of transplant,[d] n (%)						
Consolidated 1A	360 (77.6)	574 (76.8)	0.39	840 (76.9)	631 (78.5)	0.72
Consolidated 1B	83 (17.9)	123 (16.5)		202 (18.5)	133 (16.5)	
Consolidated 2	21 (4.5)	49 (6.6)		48 (4.4)	37 (4.6)	
Inactive	0 (0.0)	1 (0.1)		3 (0.3)	3 (0.4)	
Transplant characteristics						
Ex vivo machine perfusion, n (%)	11 (2.4)	13 (1.7)	0.43	11 (1.0)	8 (1.0)	0.96
Total ischemic time (hours), median (IQR)	3.6 (3.0, 4.0)	3.5 (2.9, 4.0)	0.48	3.6 (3.0, 4.1)	3.3 (2.8, 3.8)	<.0001
Gender mismatch, n (%)						
Donor female – recipient male	49 (10.6)	77 (10.3)	0.12	128 (11.7)	85 (10.6)	0.27
Donor male – recipient female	42 (9.1)	90 (12.0)		117 (10.7)	80 (10.0)	
Donor and recipient male	281 (60.6)	465 (62.2)		648 (59.3)	512 (63.7)	
Donor and recipient female	92 (19.8)	115 (15.4)		200 (18.3)	127 (15.8)	
Predicted heart mass mismatch, percent (mean ± SD)[e]	5.2 ± 19.2	5.7 ± 18.6	0.65	4.7 ±18.7	5.97 ± 19.7	0.14
Predicted heart mass mismatch ratio category, n (%)						

Type of DCU available	Hospital-based			Independent		
**Organ recovery location**	**DCU N = 464**	**Hospital N = 747**	**P-value**	**DCU N = 1,093**	**Hospital N = 804**	**P-value**
Heart undersized (donor mass <=86% of recipient)	58 (12.5)	76 (10.2)	0.26	128 (11.7)	81 (10.1)	0.30
Matched (within 87 and 114% of recipient)	296 (63.8)	469 (62.8)		701 (64.1)	508 (63.2)	
Heart oversized (donor mass >114% of recipient)	110 (23.7)	202 (27.0)		264 (24.2)	215 (26.7)	
Cytomegalovirus status, n (%)						
Donor positive / recipient negative	122 (26.3)	204 (27.3)	0.07	294 (26.9)	207 (25.7)	0.55
Donor and recipient positive	193 (41.6)	265 (35.5)		391 (35.8)	283 (35.2)	
Donor and recipient negative	67 (14.4)	103 (13.8)		191 (17.5)	147 (18.3)	
Donor negative / recipient positive	70 (15.1)	158 (21.2)		210 (19.2)	156 (19.4)	

ICU: intensive care unit, IQR: interquartile range, LVAD: left ventricular assist device, SD: standard deviation, WU: Woods units

[a]Calculated using Cockcroft-Gault Formula. [b]Includes left ventricular assist devices, biventricular assist devices, and total artificial hearts. No recipients were supported by extracorporeal membrane oxygenation at the time of transplant. [c]See Supplement Table S1. [d]Defined by UNOS status.[5] [e]Calculated using the International Society for Heart and Lung Transplantation 2019 predicted heart mass calculator.[6]

Table S6. Secondary comparisons. Secondary heart transplant recipient outcomes between organs recovered in DCUs versus hospitals

**Type of DCU available**	**Hospital-based**			**Independent**		
**Organ recovery location**	**DCU N = 464**	**Hospital N = 747**	**p-value**	**DCU N = 1,093**	**Hospital N = 804**	**p-value**
In-hospital outcomes						
Length of stay after transplant (days), median (IQR)	17 (12, 26)	16 (12, 24)	0.09	17 (12, 26)	17 (12, 26)	0.49
Acute rejection before hospital discharge, n (%)	80 (17.2)	115 (15.4)	0.40	190 (17.4)	112 (13.9)	0.04
Dialysis before hospital discharge, n (%)	68 (14.7)	97 (13.0)	0.67	164 (15.0)	121 (15.0)	0.33
One-year outcomes						
Graft survival, %[a,b]	90.9	93.3	0.77	92.3	90.1	0.24
Adjusted graft survival,[c] % (95% CI)	91.8 (89.6 – 94.0)	93.0 (91.4 – 94.6)	0.27	91.8 (90.2 – 93.3)	90.5 (88.6 – 92.3)	0.36
Long-term outcomes						
4-year restricted mean survival time, days[b]	1276	1289		1292	1265	

CI: confidence interval, DCU: donor care unit, IQR: interquartile range

[a]Estimated by the Kaplan-Meier method at 339, 300, 346, and 365 days (respectively). [b]Unadjusted. [c]Estimated from Cox proportional hazards models (see Supplement Tables S9 and S10 for covariates).

Table S7. Primary comparison. Unadjusted and adjusted Cox models for heart graft failure among deceased heart donors after brain death managed in donor care units

Covariate	Number (%) of recorded values	HR (95% CI)	aHR (95% CI)[a]
**Donor managed in independent DCU (ref. hospital-based DCU)**	1557 (100)	**0.92 (0.68 – 1.25)**	**0.79 (0.50 – 1.24)**
Donor covariates			
Donor age	1557 (100)	1.00 (0.99 – 1.02)	
Expanded criteria heart donor[2]	1557 (100)	1.16 (0.86 – 1.57)	1.18 (0.79 - 1.75)
Mechanism of death (ref. blunt injury)	1557 (100)		
Asphyxiation		1.18 (0.63 – 2.22)	
Cardiovascular		1.03 (0.61 – 1.75)	
Death from natural causes		0.81 (0.29 – 2.25)	
Drowning		0.73 (0.10 – 5.26)	
Drug intoxication		0.84 (0.56 – 1.27)	
Electrical		0.00 (0.00 -)	
Gunshot wound		1.08 (0.71 – 1.63)	
Intracranial hemorrhage or stroke		1.21 (0.76 – 1.93)	
Seizure		1.14 (0.41 – 3.15)	
Stab wound		2.06 (0.28 – 14.9)	
Other		0.45 (0.06 – 3.27)	
Left ventricular ejection fraction	1556 (99.9)	1.00 (0.98 – 1.03)	1.01 (0.98 – 1.04)
Dobutamine	1557 (100)	0.74 (0.31 – 1.80)	
Levothyroxine	1479 (95.0)	1.15 (0.87 – 1.53)	1.12 (0.75 – 1.67)
Recipient covariates			
Recipient age (10-year increments, ref. youngest)	1557 (100)	1.11 (1.01 – 1.22)	1.20 (1.03 – 1.40)
Recipient sex (ref. male)	1557 (100)	1.03 (0.76 – 1.40)	0.80 (0.52 – 1.23)
Etiology of heart failure (ref. dilated)[1]	1526 (98.0)		
Complex congenital		0.73 (0.34 – 1.55)	1.06 (0.35 – 3.22)
Hypertrophic cardiomyopathy		0.43 (0.14 – 1.34)	0.39 (0.09 – 1.70)
Ischemic cardiomyopathy		1.10 (0.41 – 2.95)	1.52 (0.41 – 5.66)
Restrictive cardiomyopathy		1.25 (0.71 – 2.20)	1.07 (0.50 – 2.29)
Arrhythmogenic		0.55 (0.08 – 3.89)	0.98 (0.11 – 9.20)
Valvular		2.21 (0.70 – 6.92)	3.01 (0.70 – 13.1)
Retransplant		1.17 (0.50 – 2.73)	1.11 (0.38 – 3.22)
LVAD at transplant (ref. no VAD)[b]	1557 (100)		
Permanent		1.56 (1.16 – 2.10)	
Temporary		0.70 (0.29 – 1.71)	
Other		1.25 (0.58 – 2.69)	
UNOS status (ref. consolidated status 2)[5]	1557 (100)		
Consolidated status 1A		1.03 (0.51 – 2.10)	1.43 (0.59 – 3.45)
Consolidated status 1B		1.42 (0.67 – 3.00)	1.83 (0.72 – 4.61)
Inactive		11.1 (2.36 – 52.6)	5.84 (0.45 – 75.7)
Pulmonary vascular resistance	1234 (79.3)	0.99 (0.90 – 1.09)	
Predicted heart mass (PHM) mismatch ratio category (ref. matched)[6]	1557 (100)		
Donor heart oversized (donor PHM >114% of recipient)		0.99 (0.71 – 1.39)	1.02 (0.65 – 1.58)
Donor heart undersized (donor PHM < 86% of recipient)		1.22 (0.81 – 1.83)	1.35 (0.77 – 2.35)
Gender mismatch (ref. donor male – recipient male)	1557 (100)		

Donor female – recipient male		1.26 (0.83 – 1.91)	
Donor male – recipient female		1.14 (0.72 – 1.80)	
Donor and recipient female		1.04 (0.72 – 1.50)	
CMV mismatch (ref. donor positive, recipient positive)	1557 (100)		
Donor negative, Recipient negative		0.62 (0.39 – 0.98)	
Donor negative, Recipient positive		0.91 (0.63 – 1.34)	
Donor positive, Recipient negative		0.86 (0.61 – 1.21)	

aHR: adjusted hazard ratio, CI: confidence interval, CMV: cytomegalovirus, DCU: donor care unit, HR: hazard ratio, LVAD: left ventricular assist device, UNOS: United Network for Organ Sharing

[a]Also stratified by transplant program and transplant year. [b]See Supplement Table S1 for classification.

Adjusted model includes 1451 cohort recipients (of 1557, 93.2%) with a complete set of donor and recipient covariates.

Table S8. Primary comparison. Characteristics and unadjusted outcomes of heart transplant recipients included, versus excluded, from the adjusted Cox graft survival model due to missingness of at least one covariate

	**Included (N = 1,451, 93.2% of primary cohort)**	**Excluded (N= 106, 6.8% of primary cohort)**
Donor characteristics		
Organ recovery location, n (%)[a]		
Independent DCU	1030 (94.2)	63 (5.8)
Hospital-based DCU	421 (90.7)	43 (9.3)
Donor age, median (IQR)	31 (24, 39)	31 (26, 39)
Male sex, n (%)	1008 (92.6)	80 (7.4)
Donor race and ethnicity, n (%)		
American Indian or Alaska Native	6 (100)	0 (0)
Asian	24 (100)	0 (0)
Black	288 (93.2)	21 (6.8)
Hispanic	239 (92.6)	19 (7.4)
Multiracial	12 (80)	3 (20)
White	882 (93.3)	63 (6.7)
Mechanism of death, n (%)		
Asphyxiation	73 (92.4)	6 (7.6)
Cardiovascular	136 (90.1)	15 (9.9)
Blunt injury	334 (91.5)	31 (8.5)
Death from natural causes	38 (97.4)	1 (2.6)
Drowning	11 (84.6)	2 (15.4)
Drug intoxication	369 (93.9)	24 (6.1)
Electrical	3 (100)	0 (0)
Gunshot wound	282 (94.0)	18 (6.0)
Intracranial hemorrhage or stroke	160 (95.8)	7 (4.2)
Seizure	24 (92.3)	2 (7.7)
Stab wound	3 (100)	0 (0)
Other	18 (100)	0 (0)
Left ventricular ejection fraction,[a] percent, median (IQR)	60 (58, 65)	61 (56, 65)
Dobutamine, n (%)	49 (94.2)	3 (5.8)
Levothyroxine,[a] n (%)	556 (97.7)	13 (2.3)
Expanded criteria donor,[2,a] n (%)	422 (96.1)	17 (3.9)
Recipient characteristics		
Transplant year, n (%)[b]		
2019	266 (96.0)	11 (4.0)
2020	365 (98.4)	6 (1.6)
2021	450 (92.4)	37 (7.6)
2022	370 (87.7)	52 (12.3)
Recipient age, years, median (IQR)[a]	55 (43, 63)	53 (37, 61)
Male sex, n (%)[a]	1027 (92.9)	79 (7.1)
Race and ethnicity, n (%)		
American Indian or Alaska Native	2 (100)	0 (0)

	Included (N = 1,451, 93.2% of primary cohort)	Excluded (N= 106, 6.8% of primary cohort)
Asian	57 (96.6)	2 (3.4)
Black	345 (93.2)	25 (6.8)
Hispanic	187 (92.6)	15 (7.4)
Multiracial	8 (80.0)	2 (20.0)
Native Hawai'ian or other Pacific Islander	2 (100)	0 (0)
White	850 (93.2)	62 (6.8)
Etiology of heart failure,[1, a] n (%)		
Complex congenital	73 (89.0)	9 (11.0)
Dilated	1166 (95.2)	59 (4.8)
Hypertrophic cardiomyopathy	52 (92.9)	4 (7.1)
Ischemic cardiomyopathy	27 (96.4)	1 (3.6)
Restrictive cardiomyopathy	71 (97.3)	2 (2.7)
Arrhythmogenic	15 (100)	0 (0)
Valvular	12 (100)	0 (0)
Retransplant	34 (100)	0 (0)
LVAD at listing, n (%)		
None	981 (93.5)	68 (6.5)
Permanent	355 (93.7)	24 (6.3)
Temporary	59 (90.8)	6 (9.2)
UNOS status,[5, a] n (%)		
Consolidated status 1A	1119 (93.3)	81 (6.7)
Consolidated status 1B	264 (92.6)	21 (7.4)
Consolidated status 2	66 (95.7)	3 (4.3)
Inactive	2 (66.6)	1 (33.3)
Pulmonary vascular resistance, WU, mean ± SD	2.3 ± 1.7	2.3 ± 1.3
Transplant characteristics		
Predicted heart mass (PHM) mismatch ratio category,[6, a] n (%)		
Donor heart oversized (donor PHM >114% of recipient)	351 (93.9)	23 (6.1)
Donor heart size match (donor PMH 86 – 114% of recipient)	923 (92.6)	74 (7.4)
Donor heart undersized (donor PHM < 86% of recipient)	177 (95.2)	9 (4.8)
Ex vivo machine perfusion, n (%)	22 (100)	0 (0)
Ischemic time, hours, median (IQR)	3.5 (3.0, 4.1)	3.6 (3.0, 4.0)
Secondary recipient outcomes		
1-year graft survival, n (%)	1132 (92)	74 (94)
4-year restricted mean survival time, days	1283	562

ICU: intensive care unit, SD: standard deviation, PHM: predicted heart mass, SD: standard deviation, SE: standard error, VAD: ventricular assist device, WU: Woods units

[a]Covariate included in multivariable Cox model. [b]Included as a stratifying variable in the multivariable Cox model.

Table S9. Secondary comparison. Adjusted Cox model for heart graft failure from deceased heart donors after brain death managed in hospital-based donor care units versus hospitals in regions with operating hospital-based DCUs

	aHR (95% CI)[a]
**Donor managed in hospital-based DCU (ref. hospital)**	**1.46 (0.93 – 2.28)**
Donor covariates	
Expanded criteria heart donor,[2] (ref. standard criteria donor)	0.87 (0.54 – 1.40)
Left ventricular ejection fraction	1.01 (0.98 – 1.04)
Levothyroxine (ref. no levothyroxine)	1.20 (0.72 – 1.99)
Recipient covariates	
Recipient age (10-year increments, ref. youngest)	1.04 (0.88 – 1.24)
Recipient sex (ref. male)	0.97 (0.55 – 1.70)
Etiology of heart failure (ref. dilated)[1]	
Complex congenital	1.72 (0.65 – 4.57)
Hypertrophic cardiomyopathy	0.80 (0.17 – 3.82)
Ischemic cardiomyopathy	0.76 (0.13 – 4.32)
Restrictive cardiomyopathy	0.47 (0.13 – 1.64)
Arrhythmogenic	0.00 (0.00 -)
Valvular	0.44 (0.04 – 4.46)
Retransplant	2.53 (0.43 – 14.9)
UNOS status (ref. consolidated status 2)[5]	
Consolidated status 1A	0.76 (0.29 – 2.00)
Consolidated status 1B	0.83 (0.29 – 2.37)
Predicted heart mass (PHM) mismatch ratio category (ref. matched)[6]	
Donor heart oversized (donor PHM >114% of recipient)	1.36 (0.79 – 2.35)
Donor heart undersized (donor PHM < 86% of recipient)	1.70 (0.84 – 3.46)

aHR: adjusted hazard ratio, CI: confidence interval, DCU: donor care unit, HR: hazard ratio, UNOS: United Network for Organ Sharing.

[a]Also stratified by transplant program and transplant year

Complete case analysis. Includes 1103 of 1211 heart donor-recipient pairs (91.1%) with hearts recovered from donation regions with a hospital-based DCU.

Table S10. Secondary comparison. Adjusted Cox model for heart graft failure from deceased heart donors after brain death managed in hospitals versus independent donor care units

	**aHR (95% CI)[a]**
**Donor managed in an independent DCU (ref. hospital)**	**0.87 (0.63 – 1.20)**
Donor covariates	
Expanded criteria heart donor.[2] (ref. standard criteria donor)	1.08 (0.77 – 1.51)
Left ventricular ejection fraction	1.00 (0.98 – 1.03)
Levothyroxine (ref. no levothyroxine)	1.44 (1.05 – 1.97)
Recipient covariates	
Recipient age (10-year increments, ref. youngest)	1.25 (1.10 – 1.42)
Recipient sex (ref. male)	0.81 (0.57 – 1.17)
Etiology of heart failure (ref. dilated)[1]	
Complex congenital	1.06 (0.45 – 2.51)
Hypertrophic cardiomyopathy	0.57 (0.17 – 1.91)
Ischemic cardiomyopathy	2.58 (1.03 – 6.47)
Restrictive cardiomyopathy	1.16 (0.59 – 2.27)
Arrhythmogenic	1.03 (0.21 – 5.19)
Valvular	1.84 (0.66 – 5.12)
Retransplant	1.62 (0.70 – 3.72)
UNOS status (ref. consolidated status 2)[5]	
Consolidated status 1A	1.29 (0.60 – 2.76)
Consolidated status 1B	1.36 (0.60 – 3.08)
Predicted heart mass (PHM) mismatch ratio category (ref. matched)[6]	
Donor heart oversized (donor PHM >114% of recipient)	1.10 (0.77 – 1.59)
Donor heart undersized (donor PHM < 86% of recipient)	1.60 (0.73 – 1.85)

aHR: adjusted hazard ratio, CI: confidence interval, DCU: donor care unit, HR: hazard ratio, UNOS: United Network for Organ Sharing.

[a]Also stratified by transplant program and transplant year

Complete case analysis. Includes 1798 of 1897 heart donor-recipient pairs (94.8%) with hearts recovered from donors in regions with an independent DCU.

Figure S1. Heart donation rates by recovery location

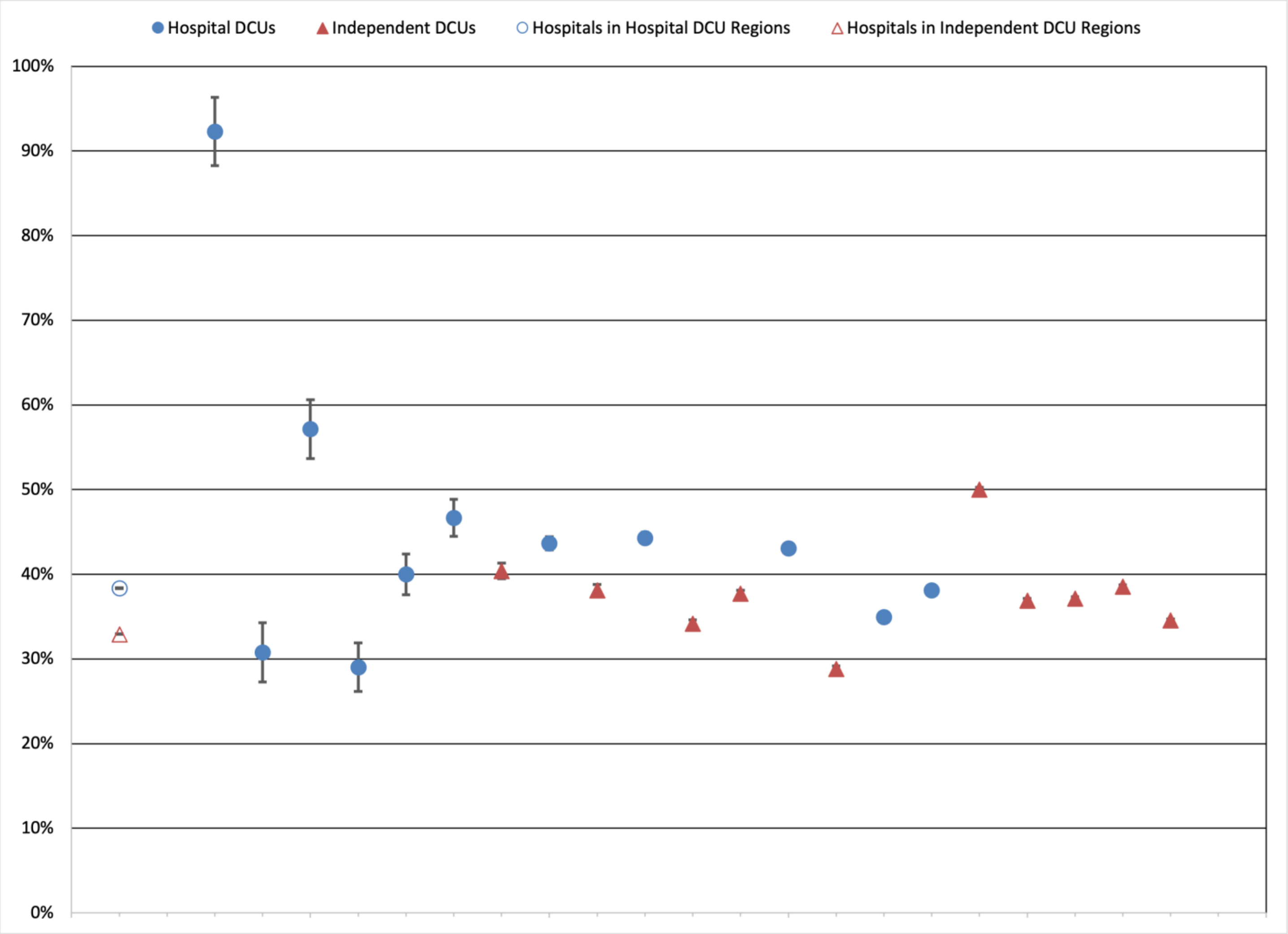

Each solid marker indicates a cohort DCU, ordered on the X-axis in ascending number of donors in the cohort. Error bars represent 95% confidence intervals for the proportion. Open markers indicate median values for donors who remained in hospitals, stratified by the type of DCU available in that organ donation region.

Y axis: Percent of donors in each DCU that donated a heart for transplant.

Figure S2. Variation in clinical heart donor management among DCUs and hospitals

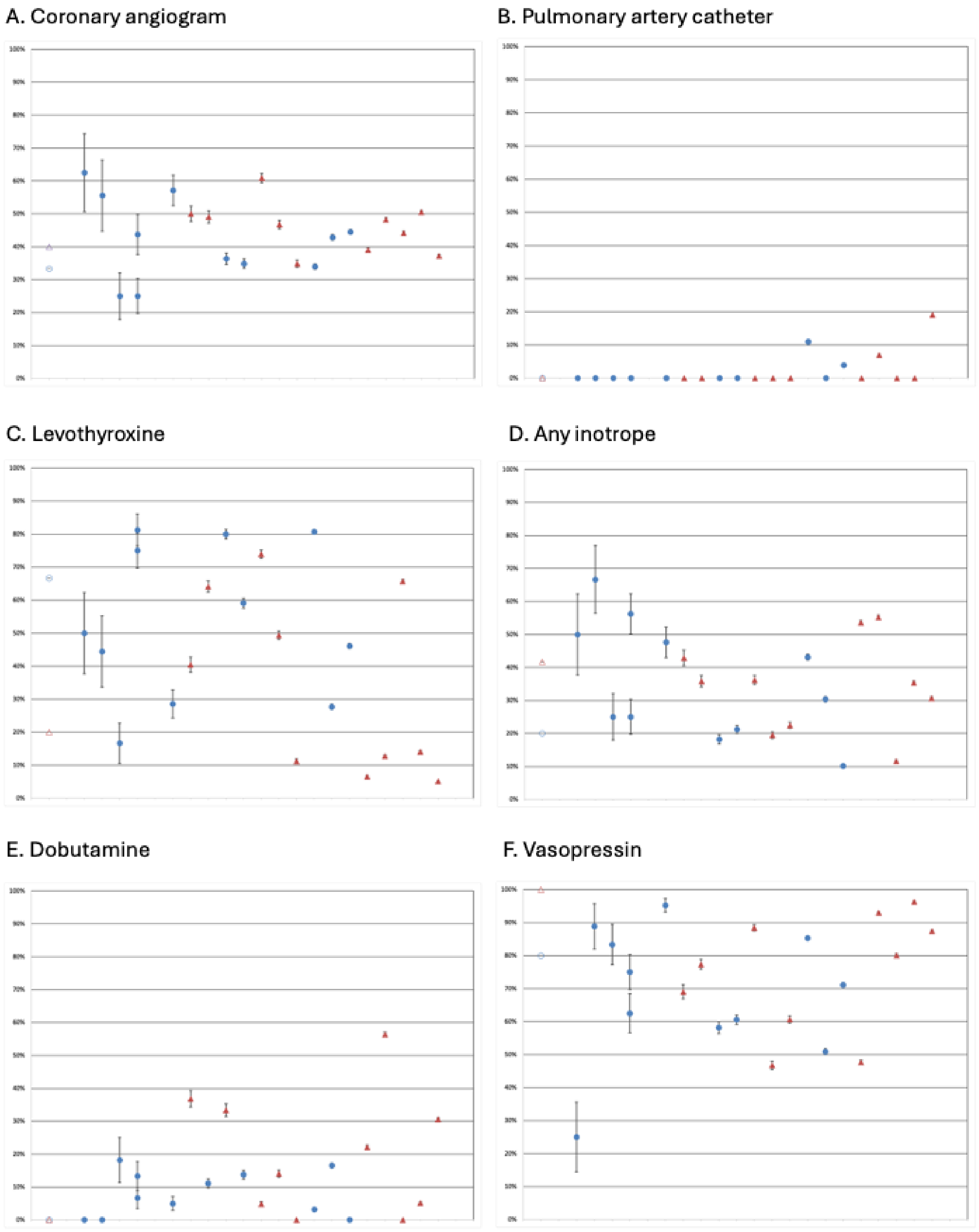

Each solid marker indicates a cohort DCU, ordered on the X-axis in ascending number of donors in the cohort. Error bars represent 95% confidence intervals for the proportion. Open markers indicate median values for donors who remained in hospitals, stratified by the type of DCU available in that organ donation region.

Y axis: Percent of donors in each DCU or hospital type that received each test or therapy during donor management.

Figure S3. Secondary comparisons. Unadjusted survival curves between recipients of hearts recovered from donor care units (DCUs) and hospitals, stratified by DCU type

Panel A. Independent DCU available

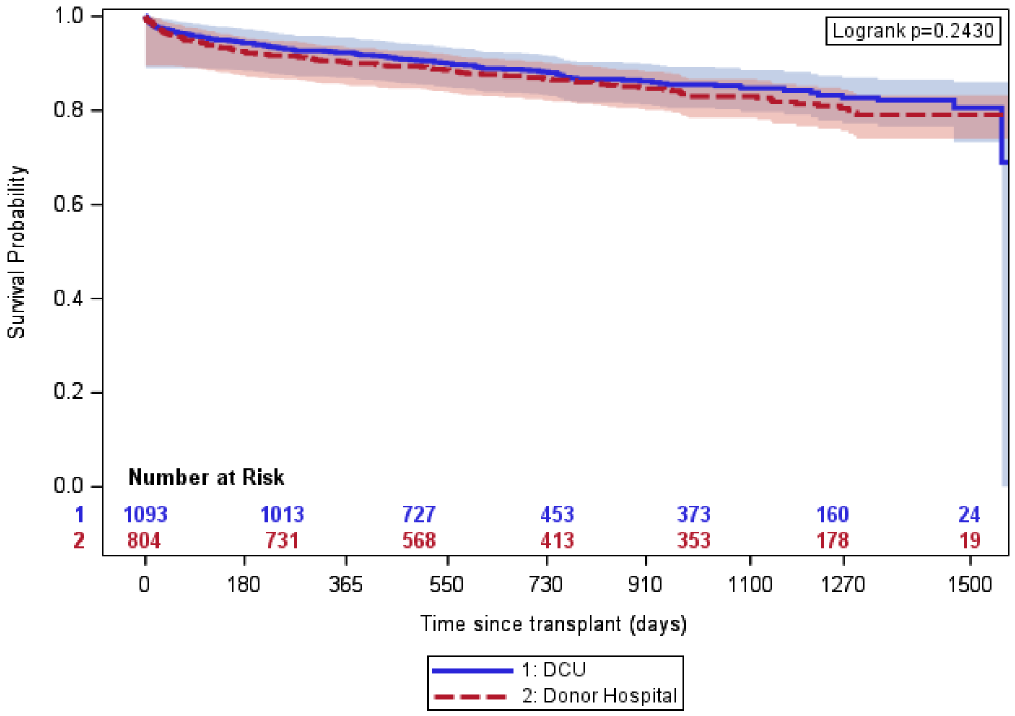

Panel B. Hospital-based DCU available

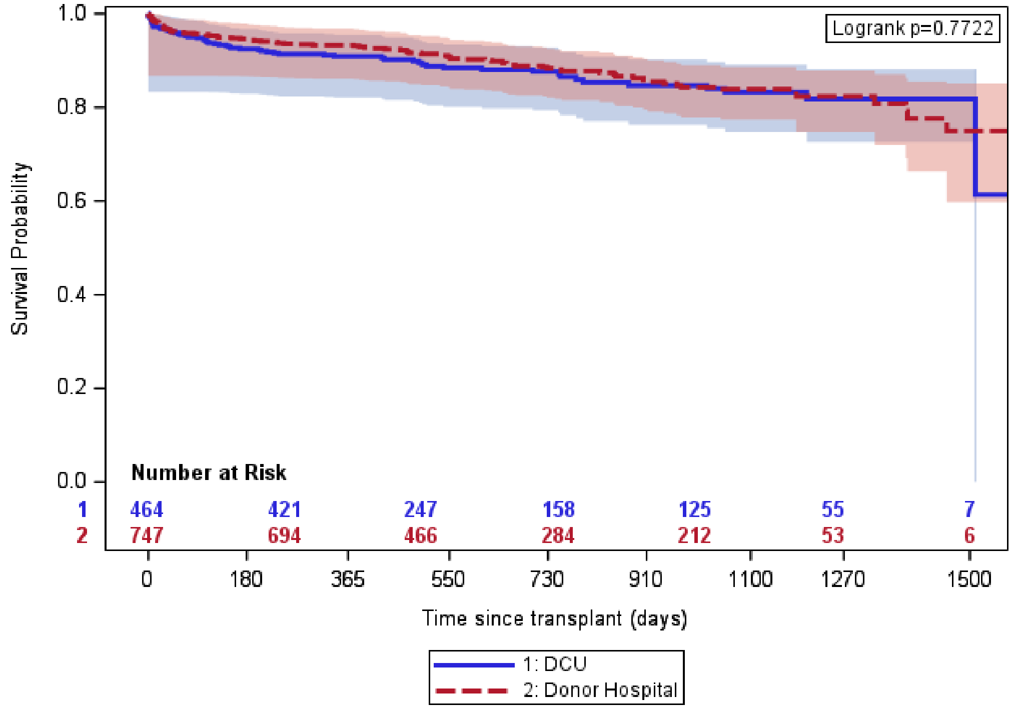

Shaded regions represent 95% confidence intervals. Donor hospitals include only hearts recovered from donors managed in acute-care hospitals in US donation regions with an operating DCU, independent or hospital-based, respectively.

Figure S4. Primary Comparison. Tests of the proportional hazards assumption for the Cox graft survival model comparing grafts recovered from hospital-based versus independent DCUs

Panel A. Baseline survivor function curves by DCU type

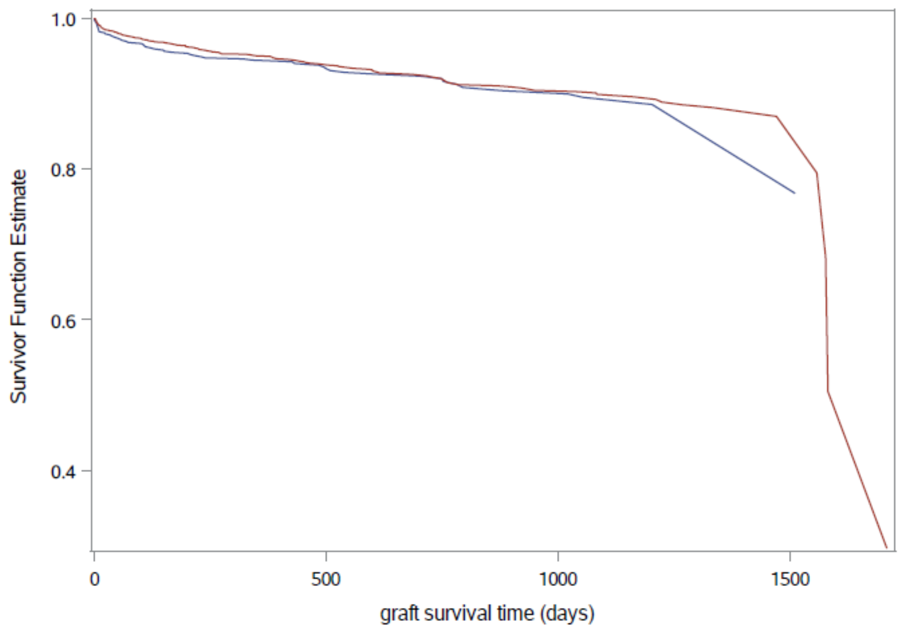

Red line: Hearts recovered from independent DCUs. Blue line: Hearts recovered from hospital-based DCUs. Baseline survivor function estimates include all model covariates with continuous variables set at 0 and categorical covariates set at the reference level.

Panel B. Log cumulative hazards function (log (-log) adjusted survival function) curves by DCU type

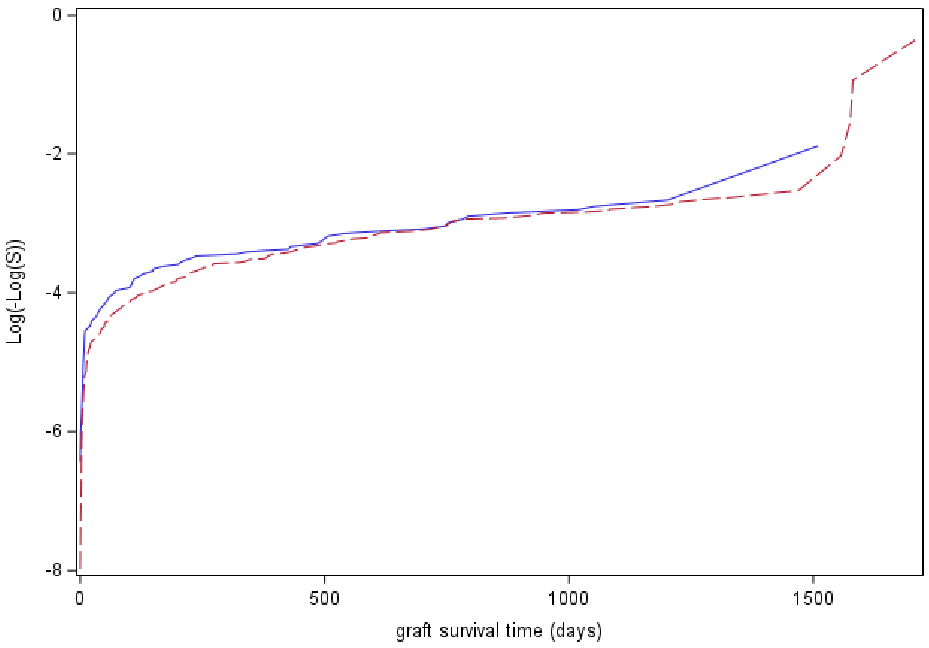

Red line: Hearts recovered from independent DCUs. Blue line: Hearts recovered from hospital-based DCUs. Survivor function estimates are generated from the adjusted Cox model with all covariates set at central values (mode or median).

Figure S5. Secondary comparisons. Adjusted survivor function curves

Panel A. Hearts recovered from DCUs vs. hospitals in donation regions with an independent DCU

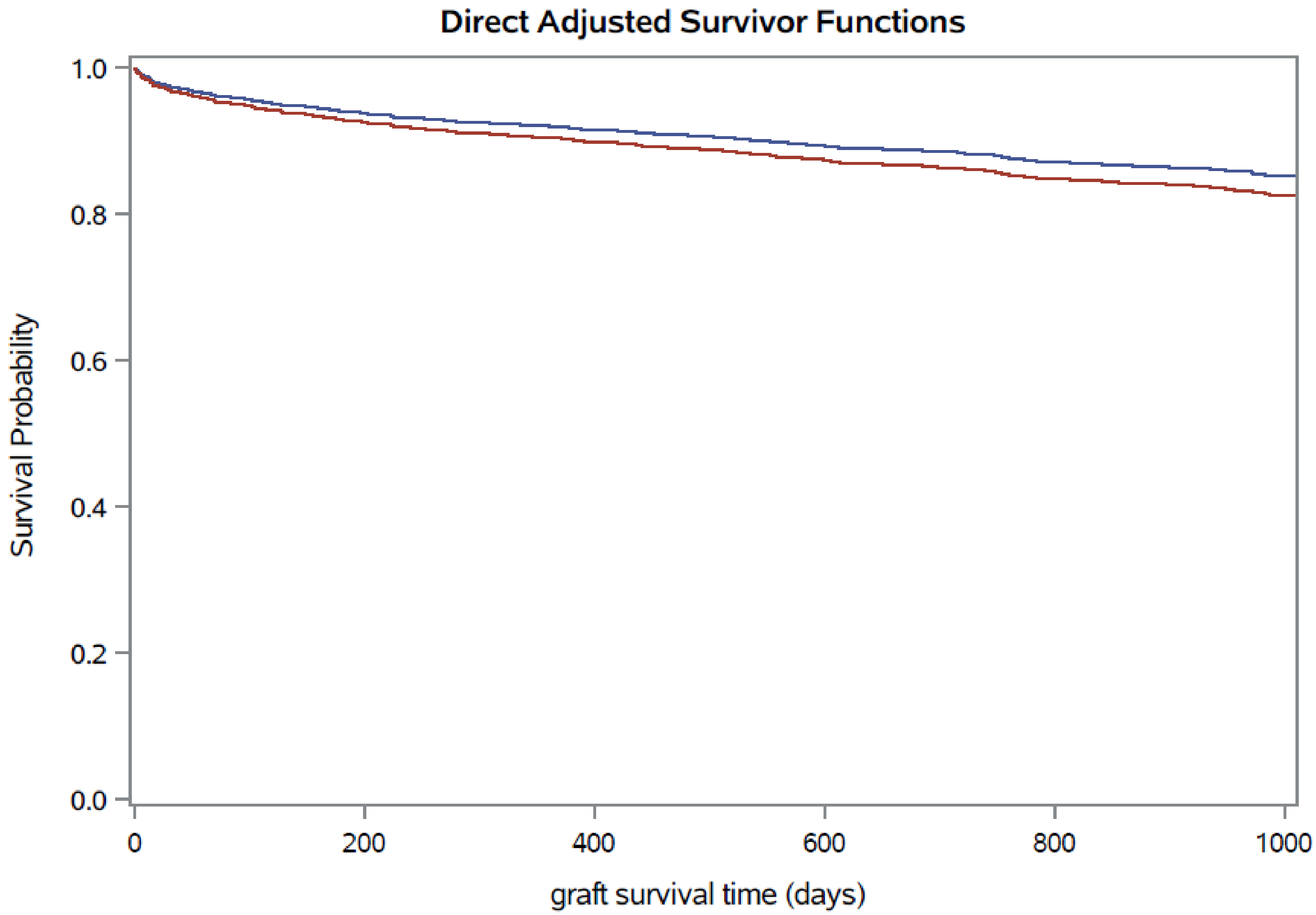

The blue line represents the estimated adjusted survival probability among hearts recovered from independent DCUs and transplanted over time. The red line represents the estimated adjusted survival probability among heart grafts recovered and transplanted from donor hospitals in donation regions with independent DCUs. Curves adjusted for transplant year, transplant program, expanded criteria heart donor status, terminal left ventricular ejection fraction, recipient age, recipient sex, etiology of heart failure, UNOS status at listing, and mismatch between predicted donor and recipient heart mass (see Table S10 in the Supplement).

Figure S5. Secondary comparisons. Adjusted survivor function curves.

Panel B. Hearts recovered from DCUs vs. hospitals in donation regions with a hospital-based DCU

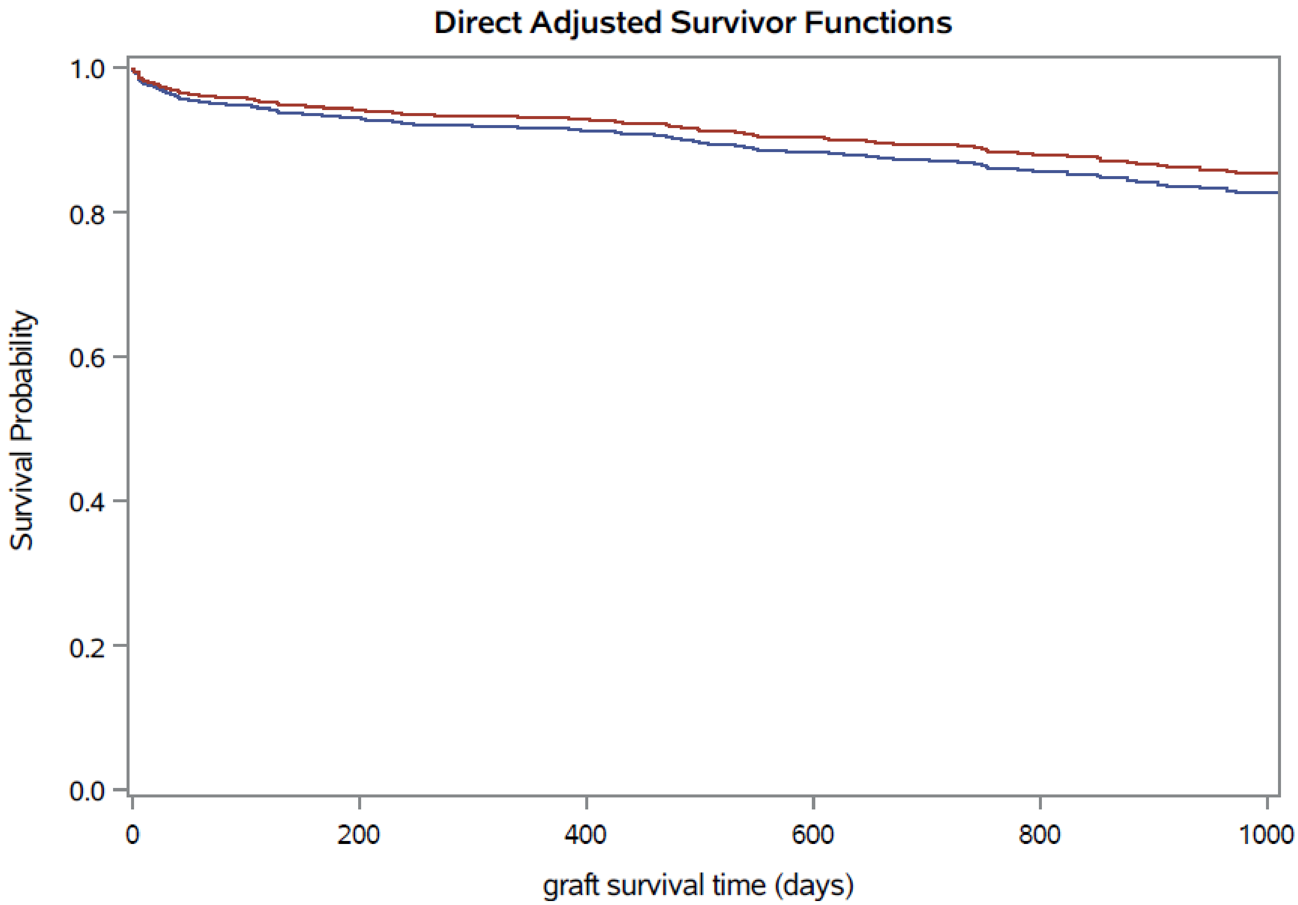

The blue line represents the estimated adjusted survival probability over time among hearts recovered from hospital-based DCUs and transplanted. The red line represents the estimated adjusted survival probability among heart grafts recovered and transplanted from hospitals in donation areas with a hospital-based DCU. Curves adjusted for transplant year, transplant program, expanded criteria heart donor status, terminal left ventricular ejection fraction, recipient age, recipient sex, etiology of heart failure, UNOS status at listing, and mismatch between predicted donor and recipient heart mass (see Table S9 in the Supplement).

Supplement references

1. Sorabella RA, Kantar A, Castillero E, Takayama H, Schulze PC, Mancini D, Naka Y, George I. Cardiac Donor Risk Factors Predictive of Short-Term Heart Transplant Recipient Mortality: An Analysis of the United Network for Organ Sharing Database. *Transplant Proc.* 2015: 47: 2944-2951. doi: 10.1016/j.transproceed.2015.10.021

2. Bakhtiyar SS, Sakowitz S, Verma A, Chervu NL, Benharash P. Expanded Criteria Donor Heart Allograft Utilization: National Trends and Outcomes. *Ann Thorac Surg* 2023; **116**: 1250-1258. doi: 10.1016/j.athoracsur.2023.09.013

3. Whitbread JJ, Etchill EW, Giuliano KA, et al. Ventricular assist devices and middle age reduce heart transplantation rates for waitlist candidates. J Card Surg. Aug 2020;35(8):1778-1786. doi:10.1111/jocs.14650

4. Scientific Registry of Transplant Recipients. *Measuring Donor Yield: How often are organs from a donor successfully transplanted?* Available at: https://www.srtr.org/about-the-data/guide-to-key-opo-metrics/opoguidearticles/donor-yield/

5. Kumar R. *United Network for Organ Sharing (UNOS) 2018 Heart Transplant Listing Guidelines Revision*. 2019. Available at: https://rk.md/2019/united-network-for-organ-sharing-unos-2018-heart-transplant-listing-guidelines-revision/

6. United Network for Organ Sharing. *Predicted Heart Mass (PHM) Match Calculator*. 2019. Available at: https://insights.unos.org/phm-calculator/
